# The role of a new insulin-like peptide in the pearl oyster *Pinctada fucata martensii*

**DOI:** 10.1038/s41598-019-57329-3

**Published:** 2020-01-16

**Authors:** Hua Zhang, Maoxian He

**Affiliations:** 1 CAS Key Laboratory of Tropical Marine Bio-resources and Ecology, South China Sea Institute of Oceanology, Chinese Academy of Sciences, Guangzhou, 510301 China; 2 Guangdong Provincial Key Laboratory of Applied Marine Biology, Guangzhou, 510301 China

**Keywords:** Peptides, Transcriptional regulatory elements, Insulin signalling

## Abstract

*Pinctada fucata martensii*, is an economically important marine bivalve species cultured for seawater pearls. At present, we know little about the molecular mechanisms of the insulin signalling pathway in this oyster. Herein, we cloned and analysed an insulin-like peptide (PfILP) and its signalling pathway-related genes. We detected their expression levels in different tissues and developmental stages. Recombinant PfILP protein was produced and found to significantly increase primary mantle cell activity and induce the expression of the proliferating cell nuclear antigen (PCNA) gene. PfILP could also regulate the 293T cell cycle by stimulating the S phase and inhibiting the G1 and G2 phases. Recombinant PfILP protein induced the expression of its signalling pathway-related genes in mantle cells. *In vitro* co-immunoprecipitation analysis showed that PfILP interacts with PfIRR. PfILP activated expression of the pfIRR protein, and also activated the mitogen-activated protein kinase (MAPK) and phosphatidylinositol 3-kinase (PI3K) pathways by stimulating phosphorylation of MAPK and AKT. Further analysis showed that PfILP up-regulated glycogen synthesis-related genes glycogen synthase kinase-3 beta (GSK-3β), protein phosphatase 1 (PP1) and glucokinase (GK) at the mRNA level, as well as the expression of the PP1 protein, and phosphorylation of GSK-3β. These results confirmed the presence of a conserved insulin-like signalling pathway in pearl oyster that is involved in cell activity, glycogen metabolism, and other physiological processes.

## Introduction

The insulin-relaxin superfamily is composed of two subfamilies: the insulin and insulin-like growth factor (IGF) subfamily, and the relaxin and insulin-like (INSL) subfamily^1^. These proteins exhibit a highly structural conservation. They share conserved cysteine residues required for the formation of disulphide bridges which are the hallmark of this superfamily^2^. The insulin-relaxin superfamily has a variety of functions; they can activate both PI3K/AKT and MAPK pathways that are composed of multiple effectors^3^. These pathways control cell growth, proliferation, differentiation and apoptosis, cell adhesion, embryo development, organ formation, bone regeneration, and other physiological processes^4–6^. Insulin is a key peptide hormone that can activate the PI3K and MAPK pathways to regulate metabolism and growth in metazoans^1,7–9^. In vertebrates, the function of insulin has been extensively studied since it is central to regulate the glycogen, fat, and protein metabolism^2,10,11^.

Insulin-like peptides (ILPs) are an ancient protein family, present in vertebrates and invertebrates, that regulate diverse physiological processes. In invertebrates, multiple ILPs have been identified, and bombyxin was the first reported ILP from the silk moth *Bombyx mori*^12,13^. An abundance of ILPs have since been identified in various invertebrates species, including the branchiopod crustacean *Daphnia pulex*^14^, parasitic platyhelminths^15^, the nematode *Caenorhabditis elegans*^16^ and various molluscs^17,18^, but primarily in arthropods^19^.

In insects, the structure and functiona of ILPs is similar to vertebrate insulins and relaxin^20^. In addition to sharing similar functions with vertebrate peptides, insect ILPs also regulate neurotransmitters and growth factors^19,21,22^. Signalling of ILPs is broadly studied in the fruit fly *Drosophila melanogaster*. Eight genes encoding ILPs (ILP1-8) in its genome were found^20,23,24^. These ILPs are coupled to insulin-like receptor, and then activate downstream signal network^25,26^. Besides, Dilp8 can bind GPCR-namely LGR3 and LGR4 to mediate the regeneration checkpoint developmental delay and growth coordination in *D*.*melanogaster*^27,28^. Insulin/ILP signalling pathways then regulates cell proliferation, differentiation and growth of organisms^29–31^.

In molluscs, ILPs have been identified in the *Lymnea stagnalis*^32,33^, *Aplysia californica*^17^, *Anodonta cygnea*^34^, and the *Sinonovacula constricta*^13^. However, most previous studies have only deal with the primary characterization of gene products and that more functional data are needed. Regarding ILP receptors, a unique pfIR receptor (PfIRR) was identified in *P*. *fucata martensii* and found to be mainly expressed in the testis and adductor muscle^35^. Moreover, an IGF binding protein gene (*Pfigfbp*) from *P*. *fucata martensii* was characterised, and found to be transcribed mainly in the foot^36^. However, no insulin-like analogues or peptides were found in *P*. *fucata martensii*, and effectors of the insulin-like signalling pathway remain poorly documented. In addition, it is unknown whether PfILPs has similar functions to vertebrates insulins or IGFs. Furthermore, whether PfILP can act like vertebrate insulin/IGFs and activate similar downstream signalling pathways in *P*. *fucata martensii* remains unknown.

To investigate these questions, in the present work we identified the first ILP and six effectors in the pearl oyster *P*. *fucata martensii*. We analysed their phylogenetic relationships with other homologous proteins and determined their spatio-temporal expression by quantitative real-time PCR (qPCR). We probed the interactions between recombinant PfILP and pfIRR, and the effects of PfILP-mediated pfIRR stimulation in the activation of the MAPK and PI3K signalling cascades in primary *P*. *fucata martensii* mantle cells. We further examined the biological activity of PfILP using *in vitro* phosphorylation assays and investigated its modulatory roles in glycogen metabolism and the cell cycle.

## Results

### Identification of seven ILP signalling pathway genes

**Figure 1:**
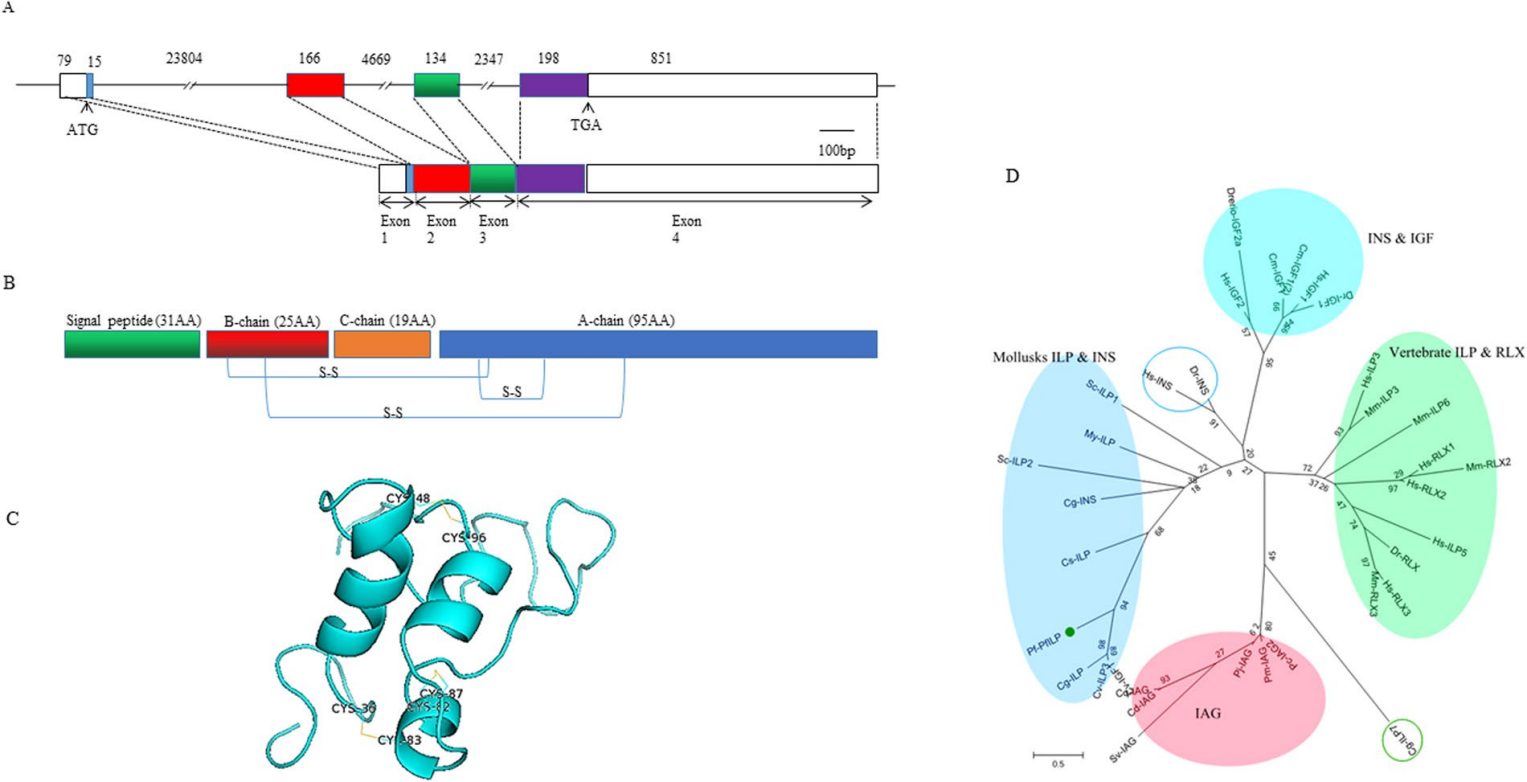
Basic information of PfILP. (**A**) Schematic diagram of the *PfILP* gene. The gene contains four exons and three introns. (**B**) Schematic representation of the predicted primary structure of the PfILP protein. (**C**) Predicted three-dimensional model of PfILP based on human insulin-like growth factor 1 (IGF1; PDB id 2gf1). Conserved cysteine residues and the resulting disulphide bridges are marked. The model was constructed using Swiss-Model and edited using PyMOL Viewer. (**D**) Maximum likelihood phylogenetic tree of PfILP from *P*. *fucata martensii* with homologs from other species. PfILP from *P*. *fucata martensii* is marked with a green circle. Numbers at tree nodes indicate the bootstrap percentage after 1000 replicates.

Seven potential effectors related to ILP signalling (based on sequence homology)- *Pfirs1*, *Pfpik3r1*, *Pfakt*, *Pfsos2*, *Pfrap-1*, *Pfraf* and *PfILP*- were identified from *P*. *fucata martensii*(Supplementary Table S1). BLASTp analyses showed high sequence homology between these six genes (except the PfILP) and genes known to be involved in the insulin signaling pathway (Supplementary Table S1). Sequence alignment demonstrated the levels of high identity; From an evolutionary perspective, six ILP signalling pathway genes are thought to have evolved from an ancestral gene, respectively, which explains the common features (Supplementary Figs. 1–6). For PfILP genes, it has four exons and three introns in genomic (Fig. 1A). PfILP protein comprises a signal peptide followed by a B-chain, a connecting peptide (C-peptide), and an A-chain (Fig. 1B). PfILP includes six cysteines in the A and B chain, allowing the formation of disulphide bridges, and two putative disulphide bonds were assumed to form two inter-chain bridges across the two chains, and one intra-chain bond on the A-chain (Fig. 1B) that is essential for tertiary folding. The 3D structure of PfILP is consistent with this phenomenon (Fig. 1C). Multiple sequence alignment of PfILP showed that their sequences are poorly conserved, especially among invertebrates, except for the six cysteine sites, which are strictly conserved (Supplementary Fig. 7). The GenBank number is in Supplementary Table S2. The phylogenetic tree shows that PfILP sequence identified in *P*. *fucata martensii* is orthologous to the previously identified mollusks ILPs in *Crassostrea gigas*, *Crassostrea virginica*, *Mizuhopecten yessoensis*, *Sinonovacula consricta* (Fig. 1D). The GenBank number is in Supplementary Table S3. The identified ILP signalling-related components provide valuable information for future studies of this pathway in *P*. *fucata martensii*.

### The expression patterns of seven ILP signalling pathway genes in different tissues and developmental stages

**Figure 2:**
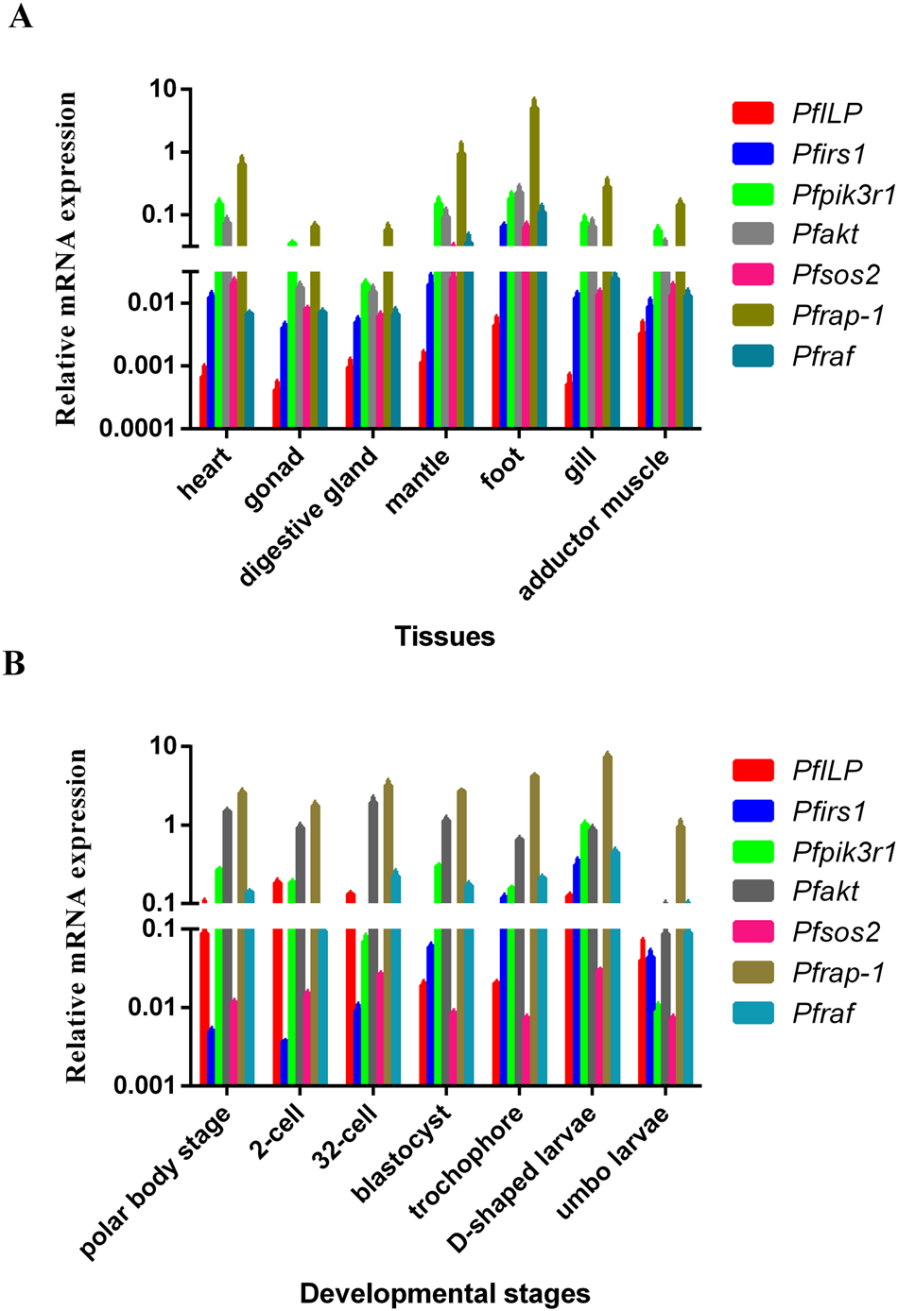
Expression analyses of seven ILP signalling pathway genes. Expression levels of *PfILP*, *Pfirs1*, *Pfpik3r1*, *Pfakt*, *Pfsos2*, *Pfrap-1* and *Pfraf* in healthy tissues (**A**) and developmental stage (**B**) of *P*. *fucata maretnsii* measured by real-time quantitative real-time PCR. The 18S rRNA gene was used as an internal control and calculated using the 2^−ΔΔCT^ method. Each vertical bar represents the mean ± SEM (n = 3).

Seven tissues from pearl oysters or different developmental stages were selected to determine the basal transcriptional levels by real-time PCR. Most of them are highly expressed in the foot or in D-shaped larvae whereas, in contrast in adductor muscle or 32-cells embryos there is apparently contrasting expression patterns (Fig. 2). The results showed that the expression patterns of the seven genes in tissues or developmental stages were totally same, which suggests that they might be involved in same physiological activities of in *P*. *fucata martensii*.

### Production of recombinant PfILP

**Figure 3:**
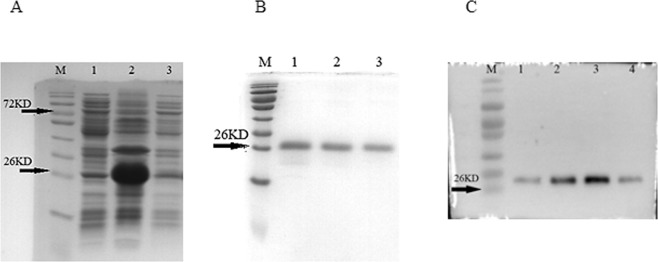
Purification of recombinant PfILP. **(A**) SDS-PAGE (12%) analysis of recombinant PfILP after 1 mM IPTG induction. Lane 1, soluble fraction after ultrasonication; Lane 2, insoluble fraction after ultrasonication precipitation; Lane 3, total protein from induced E. coli cells harbouring pET28a. (**B**) Inclusion body proteins were washed, dissolved, refolded and purified. Lanes 1–3, recombinant PfILP eluted with 250 mM imidazole. (**C**) Purified PfILP blotted with a rabbit anti-His IgG antibody (lane 1–4). Each set of bands for each protein is from the same gel.

The expression vector containing a cDNA encoding the mature PfILP polypeptide (no signal peptide) fused to a His-tag was constructed and transformed into *E*. *coli Transetta* (DE3) cells, and expression of recombinant PfILP protein was induced by IPTG. The results showed that most of the protein was present in insoluble inclusion bodies (Fig. 3A). Negative control cells containing vector alone did not yield overexpression bands. The His-tagged PfILP protein was retrieved from inclusion bodies via solubilisation before purification. Refolding PfILP protein sample was filtered to remove impurities from the resin by repetitive washing. And then a single protein band of 26 kDa was greatly enriched in the final column eluate (Fig. 3B). Subsequent immunoblotting performed with anti-His antibody revealed that the purified protein was specifically labelled (Fig. 3C).

### Cell viability following treatment with recombinant PfILP

**Figure 4:**
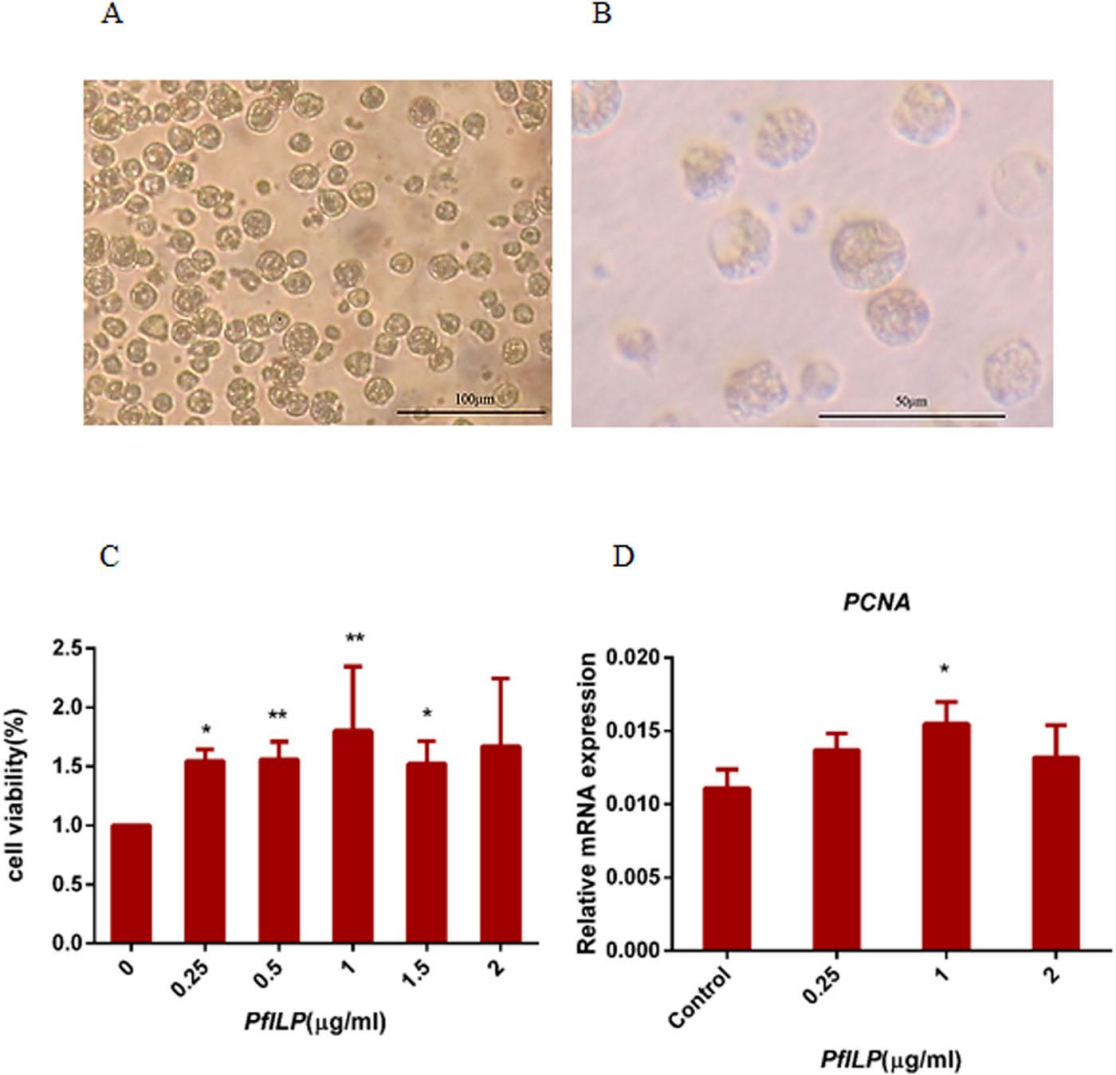
Effects of PfILP on cell activity and PCNA gene expression. (**A**) Living conditions of primary *P*. *fucata martensii* mantle cells over 2 weeks. At day 15, more cells migrated; (**B**) Cell suspension stained with trypan blue. (**C**) Cells were treated with different doses of PfILP protein. *significantly different from controls at p < 0.05; **extremely significantly different from controls at p < 0.01 (n = 3). (**D**) Analysis of PCNA transcript levels following treatment with different concentrations of PfILP. *p < 0.05, **p < 0.01.

A large quantity of cells were migrated out from explant at day 7. Cells almost covered plates at 2 weeks (Fig. 4A). Trypan blue staining demonstrated that more than 90% of cells survived up 2 weeks (Fig. 4B). The mitogenic effect of PfILP was examined by measuring the cell viability of primary cells using a Cell Counting Kit-8. The results clearly showed that PfILP improved mantle cell viability at a concentration of 0.25 μg/ml till top at 1 μg/ml (Fig. 4C). Additionally, the PCNA gene was up-regulated maximally by PfILP at a dose of 1 μg/ml (Fig. 4D).

### PfILP regulates the expression of pfIRR

**Figure 5:**
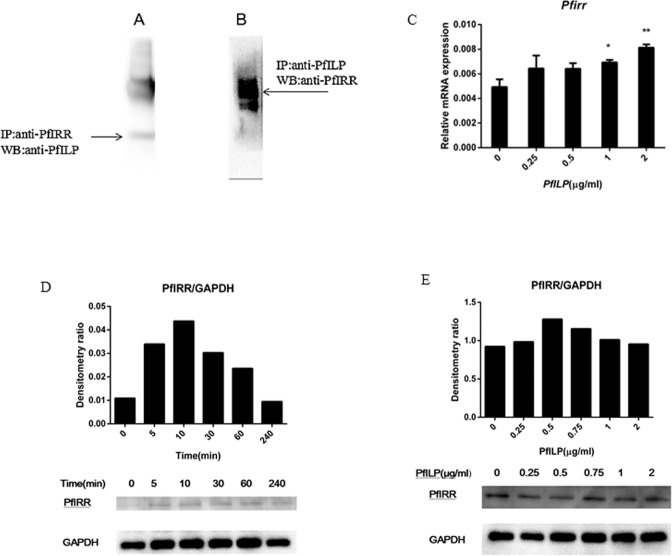
Effects of PfILP on PfIRR. **(A**,**B**) Co-IP analysis of the interaction between PfILP and PfIRR *in vitro*; (**C**) Quantitative relative expression of *Pfirr* in mantle cells after a 24 h incubation with various concentrations of PfILP. Each bar represents the mean ± S.E.M. (n = 3). *p < 0.05, **p < 0.01. (**D**,**E**) Western blotting analysis of pfIRR protein expression following treatment with PfILP for different timepoints and at different concentrations. GAPDH was used as an internal reference. Each set of bands for each protein is from the same gel.

To verify the interaction between PfILP and pfIRR, *in vitro* Co-IP analysis was performed using *P*. *fucata martensii* mantle cells. The results showed that PfILP was co-immunoprecipitated by pfIRR (Fig. 5A), and pfIRR was co-immunoprecipitated by PfILP (Fig. 5B). Additionally, we investigated the expression of pfIRR in mantle cells following treatment with PfILP. The *pfirr* transcript was significantly up-regulated following treatment with 1.0 μg/ml or 2.0 μg/ml PfILP (Fig. 5C). The pfIRR protein expression was examined by determining the ratio of pfIRR/GAPDH. As shown in Fig. 5D, PfILP induced pfIRR protein expression within 5 min, levels peaked at 10 min, and induction lasted for 1 h. When treated for 30 min, PfILP induced pfIRR expression at a dose of 0.25 μg/ml, expression was maximal with a dose of 0.5 μg/ml (Fig. 5E) and induction was observed up to a dose of 2.0 μg/ml.

### PfILP activates MAPK and PI3K/Akt signalling pathways

**Figure 6:**
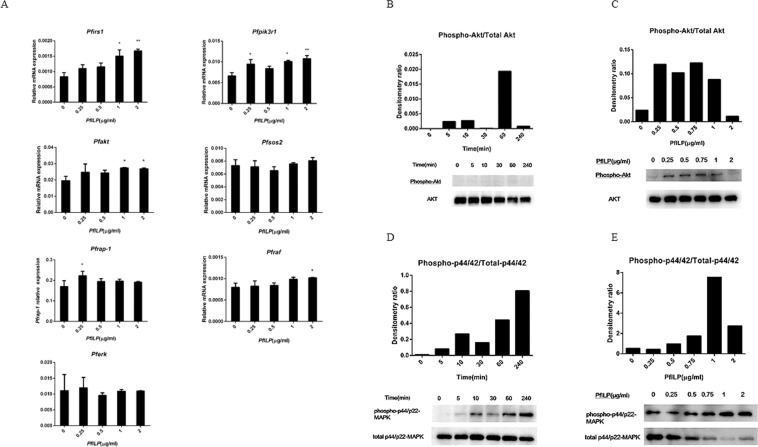
Effects of PfILP on ILP signalling pathway genes and protein expression in cultured primary mantle cells. (**A**) Quantitative relative expression of *Pfirs1*, *Pfpik3r1*, *Pfakt*, *Pfsos2*, *Pfrap-1*, *Pfraf* and *Pferk* in mantle cells after a 24 h incubation various concentrations of PfILP. Each bar represents the mean ± S.E.M. (n = 3). *p < 0.05, **p < 0.01. (**B**–**E**) Mantle cells were analysed by western blotting to detect the phosphorylation of Akt/PKB at residue T308, and p44/42 MAPK, as well as the amounts of Akt/PKB and p44/42 MAPK. Each set of bands for each protein is from the same gel.

To study the effects of PfILP on activation of the MAPK and PI3K signal transduction pathways, the seven genes (*Pfirs1*, *Pfpik3r1*, *Pfakt*, *Pfsos2*, *Pfrap-1*, *Pfraf* and *Pferk*) associated with the insulin-like signalling pathway of *P*. *fucata martensii* were examined. Additionally, phospho-p44/42 MAPK, p44/42/MAPK, phospho-Akt and Akt also were examined. As the treatment concentration of PfILP increased, *Pfirs1*, *Pfpik3r1* and *Pfakt* transcripts were upregulated, and levels peaked at a dose of 2.0 μg/ml. By contrast, *Pfsos2* and *Pferk* transcripts were not significantly changed. The *Pfrap-1* transcript was upregulated at a dose of 0.25 μg/ml, and the *Pfraf* transcript was upregulated at a dose of 2.0 μg/ml (Fig. 6A).

Phosphorylation of Akt was examined by determining the ratio of phosphorylated to total Akt. As shown in Fig. 6B, PfILP induced the phosphorylation of Akt (on residue T308) from 5 min to 240 min, and phospho-Akt levels peaked at 60 min. PfILP increased Akt (T308) phosphorylation with increasing dose from 0.25 μg/ml to 1.0 μg/ml, but this declined at a dose of 2.0 μg/ml (Fig. 6C). PfILP also induced MAPK phosphorylation within 5 min, and the induction lasted for 240 min (Fig. 6D). Again, activation time-dependently increased from 0 to 240 min. When treated for 30 min, PfILP dose-dependently induced phosphorylation of p44/42 MAPK at a concentration ranging from 0 to 1.0 μg/ml (Fig. 6E). These results clearly show that recombinant PfILP activates both MAPK and PI3K/Akt signalling pathways.

### PfILP influences the expression of glycogen-related genes and proteins

**Figure 7:**
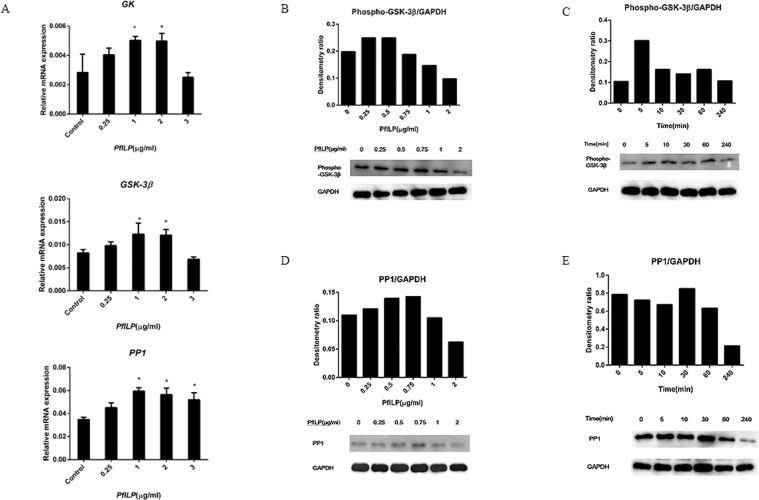
Effects of PfILP on glycogen metabolism-related genes and protein expression in cultured primary mantle cells. (**A**) Quantitative relative expression of *GK*, *GSK-3β* and *PP1* in mantle cells after a 24 h incubation with PfILP at various concentrations. Each bar represents the mean ± S.E.M. (n = 3). *p < 0.05, **p < 0.01. (**B**–**E**) Phospho-GSK-3β and PP1 protein levels were analysed by western blotting following treatment with PfILP at different concentrations or for different durations. GAPDH was used as an internal reference. Each set of bands for each protein is from the same gel.

To investigate the participation and conservation of carbohydrate/glycogen metabolism in response to PfILP induction, we analysed three glycogen-related genes involved in PfILP signalling. The results showed that the relative expression levels of *GSK-3β*, *GK* and *PP1* were significantly increased in mantle cells after PfILP stimulation (Fig. 7A).

Levels of phospho-GSK-3β and PP1 proteins were measured by western blotting, and phospho-GSK-3β levels increased gradually and reached a maximum at a dose of 0.5 μg/ml PfILP (Fig. 7B). With increasing PfILP concentration, the phosphorylation level of GSK-3β began to dose-dependently decline from 0.5 μg/ml to 2.0 μg/ml PfILP. PfILP induced GSK-3β phosphorylation within 5 min, and the induction lasted for 1 h (Fig. 7C). When treated for 30 min, PP1 expression dose-dependently increased from 0.25 μg/ml to 0.75 μg/ml. However, PP1 expression was decreased at a dose of 1.0 μg/ml and 2.0 μg/ml (Fig. 7D). At the same concentrations, PfILP time-dependently decreased PP1 expression from 0 to 240 min, except for the 30 min timepoint (Fig. 7E).

### Recombinant PfILP affects cell cycle progression

**Figure 8:**
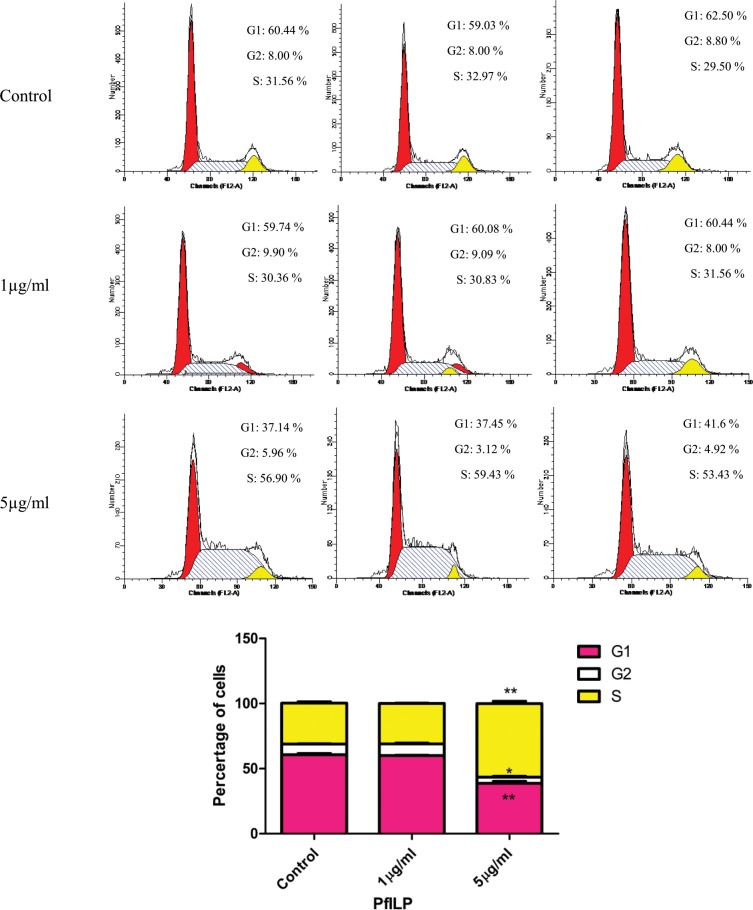
Flow cytometry analysis of the effect of PfILP on the cell cycle of 293 T cells. Cells were treated with different doses of PfILP for 36 h, and the cycle distribution was analysed by FACS analysis. Each bar corresponds to the mean ± SEM for three independent experiments. *p < 0.05, **p < 0.01.

Due to the weak proliferation of mantle cells, 293 T cells were used to investigate the effect of PfILP on cell cycle phase distribution. Cells were treated with recombinant PfLIP protein, stained with FxCycle PI/Rnase Staining Solution, and analysed by FACS. As shown in Fig. 8, a 5 μg/ml dose of recombinant PfLIP protein caused a significant decrease in the number of cells in G1 and G2 phases, with a corresponding significant accumulation of cells in S phases. These results indicate that PfILP promotes the transition of 293 T cells from G1 to S, and decreases the number of cells in G2. Thus, PfLIP promotes cell cycle progression.

## Discussion

The insulin/insulin-like signalling pathway is evolutionarily conserved in invertebrate and vertebrate animals^37,38^. Several key insulin pathway components including *PfILP*, *Pfirs1*, *Pfpik3r1*, *Pfakt*, *Pfsos2*, *PfRap-1* and *PfRaf* have been cloned from *P*. *fucata martensii*. PfILP shares the typical characteristics of ILP proteins, including six conserved cysteine residues that form disulphide bridges, and a similar 3D structure (Fig. 1). Previous studies showed that the insulin-relaxin superfamily have a similar modular organisation as their precursor, including an N-terminal signal peptide, A, B and C domains^2,13^. Post-translational modification leads to cleavage of the signal peptide and the C-peptide, resulting in a mature hormone consisting of the A and B chains. The six conserved cysteine residues in ILPs form one intra-chain (within the A-chain) and two inter-chain disulphide bridges that play a key role in protein function by cross-linking peptides^19,32^. Herein, six potential effectors of signalling, sharing varying degrees of conservation, were investigated. In vertebrates, these effectors are related to the MAPK pathway (*Pfsos2*, *PfRap-1* and *PfRaf*) or the PI3K pathway (*Pfirs1*, *Pfpik3r1* and *Pfakt*), suggesting both pathways may be conserved in molluscan species.

Analysis of the expression of *Pfirs1*, *Pfpik3r1*, *Pfakt*, *Pfsos2*, *Pfrap-1*, *Pfraf* and *PfILP* revealed that they were expressed during different developmental stages and in various tissues (Fig. 2). These results indicate that these proteins encoded by these genes may have broad functions. ILP signalling pathway genes display similar expression patterns in oyster tissues. It should be noted that PfILPs (including *Pfirs1*, *Pfpik3r1*, *Pfakt*, *Pfsos2*, *PfRap-1* and *PfRaf*) were highly expressed in the foot. In oysters, the neuroendocrine system is relatively developed in the foot. In mollusc species, growth, reproduction, and their associated metabolic processes are known to be subjected to neuroendocrine control mechanisms, and nervous ganglia are crucial centres for the production of regulatory molecules^3^. However, they were expressed primarily in D-shape larvae in the present study. The D-shape larval stage is a period in which embryonic development and organ formation is relatively fast. Furthermore, the D-shape larval stage is crucial for soft tissue and shell growth. High expression of ILP signalling pathway genes during this developmental stage implies that they may play a crucial role.

In this study, we explored a mantle primary cell culture as an *in vitro* model to investigate the effect of the insulin-like peptides PfILP on *P*. *fucata martensii* mantle cell development and metabolism. The recombinant mature PfILP peptide (without the signal peptide) increased mantle cell viability (Fig. 4A), and upregulated the PCNA gene. This phenomenon show that PfILP may promote cell proliferation. In different gastropod species, neuroendocrine factors participate in soft growth and shell growth by stimulating cell proliferation and protein synthesis^39–43^.

In brain, ILPs are specifically synthesised and released into the hemolymph, and then transported to target cells, where they interact with insulin receptors, triggering downstream signalling pathways^44–46^. Using Co-IP assays, PfILP and pfIRR were shown to interact with each other (Fig. 5A,B). Moreover, we investigated if the binding of PfILP to ILP receptors (pfRR) could activate the receptors and elicit downstream signalling cascades in *P*. *fucata martensii*. Early research shows that the activation of IGF receptors can cause the MAPK and PI3K/Akt pathways cascades^47,48^. In our previous study, human IGF-1 was shown to interact with *P*. *fucata martensii* pfIRR and to activate MAPK and PI3K/Akt pathways in *P*. *fucata martensii* oocytes^49^. Furthermore, in *Sparus aurata*, insulin signalling pathway-related transcripts were affected by IGF-I or IGF-II^50^. In our study, most ILP signalling pathway-related genes in *P*. *fucata martensii* was up-regulated in response to PfILP. Moreover, we demonstrate that PfILP also induced the expression of pfRR (Fig. 5C–E) and activated PI3K/Akt and MAPK pathways by stimulating phosphorylation of Akt and MAPK, although their threshold concentrations and durations differed (Fig. 6). Previous studies reported that Akt activation stimulates myogenic differentiation, whereas MAPK activation stimulates mitogenesis^48,51,52^. These results showed that PfILP can activate downstream intracellular signalling pathways in *P*. *fucata martensii*.

Early research found that exogenous insulin regulate glycogen metabolism in invertebrates such as the white shrimp *Penaeus vannamei*^53^ and the lobster *Panulirus argus*^54^. HrIGF-I through coupled PfIRR to activate the MAPK and PI3K signaling pathways, and then regulate glycogen metabolism in *P*. *fucata martensii*^51^. GSK3 can regulate the activity of GS to synthesis Glycogen^55,56^. Insulin can inhibit GSK3 to activate GS by, and then activate PP1^57^. PP1 is an important regulator in blood glucose levels as well as glycogen metabolism in the liver^57^. And GK act as a glucose sensor by catalysing the phosphorylation of glucose to glucose-6-phosphat in carbohydrate metabolism^58^. In our work, PP1, GSK-3β and GK mRNA levels were upregulated significantly by PfILP (Fig. 7). These results suggest that PfILP regulates glycogen metabolism pathways like vertebrate ILP. Furthermore, cell cycle analysis showed that PfILP influenced 293 T cell growth by promoting cell cycle progression (Fig. 8).

In summary, our discovery of PfILPs and six effectors in a mollusc species strengthens the hypothesis that the insulin signaling pathway has a common ancestor between vertebrates and invertebrates. Our findings can help us better understand the regulatory mechanisms and biological roles of insulin-like signalling pathways in *P*. *fucata martensii* and other invertebrates.

## Materials and Methods

Pearl oysters (2-year-old) were obtained from the Daya Bay Marine Biology Research Stations of the Chinese Academy of Sciences (Shenzhen, Guangdong, P.R. China) and maintained in an aerated indoor cement ponds for 1 week under controlled temperature (20 ± 2 °C) and fed *Chlorella vulgaris* every day. Animal experimentation in this study was approved by Experimental Animal Ethics of Chinese Academy of Sciences.

### RNA extraction

Total RNA were extracted using a TRIzol (Magen, Guangzhou, China). The integrity of RNA was determined by a 1% agarose gel electrophoresis. The quality and quantity of RNA were measured with a Quawell Q5000 (Thermo, California, USA). First-strand cDNA synthesis was performed using a SMART RACE cDNA Amplification Kit (Clontech, Palo Alto, CA, USA).

For analysis of gene expression in different tissues, mantle, digestive gland, gonad, adductor muscle, foot, heart, and gill were extracted as described above. The embryo development stages samples-the polar body, 2-cell, 32-cell, blastocyst, trochophore, D-shaped larvae, and umbo larvae stage were also extracted as above. Nine pearl oysters were randomly divided into three replicates, and equal quantities (500 ng) of total RNA from different tissues were reverse-transcribed into cDNA templates for ReverTra Ace qPCR using RT Master Mix with gDNA Remover (Toyobo, Osaka, Japan) according to the manufacturer’s instructions.

### Cloning of ILP signalling elements

To clone ILP signalling elements from *P*. *fucata martensii*, multiple pairs of primers (Supplementary Table S4) were designed according to partial fragments from the transcriptome and used to amplify full-length cDNAs. PCR products were cloned into the pGM-T Fast Vector (TIANGEN, Beijing, China) and the resulting constructs were transformed into *Escherichia coli* DH5α cells for DNA sequencing.

### Sequence characterisation and phylogenetic analysis

The cDNA sequences were evaluated by TBLASTX (NCBI; http://www.ncbi.nlm.nih.gov/blast/). The PfILP genomic sequence was obtained by searching genomic database (https://www.ncbi.nlm.nih.gov/genome/?term=Pinctada+fucata+martensii) using the full-length cDNA sequence. The genomic and cDNA sequences were then compared to derive the genomic structure of this gene (https://www.ncbi.nlm.nih.gov/sutils/splign/splign.cgi?textpage=online&level=form).DNAStar software was used to predicte the open reading frame (ORF). The simple modular architecture research tool (SMART; http://marinegenomics.oist.jp/pearl/viewer?-project_id=36) was used to analyse the amino acid structure. ExPASy tools (http://cn.expasy.org/tools/pi_tool.html) was used to analyse the molecular weight and isoelectric point (pI). Signal sequence prediction was performed using the SignaIP4.0 Server (http://www.cbs.dtu.dk/services/SignalP/). The tertiary structure of ILPs was predicted using SWISS-MODEL (https://www.swissmodel.expasy.org/interactive) and edited using PyMOL Viewer (www.pymol.org). Multiple sequence alignment was performed using CLC Main Workbench 7.7.3 software (CLC Bio, Aarhus, Denmark) to highlight regions of conservation. MEGA 6.0 software with the maximum likelihood method (1000 bootstrap replicates) was used to construct the phylogenetic trees^59^.

### Real-time quantitative PCR (qPCR) analysis

Gene expression levels was determined by qPCR using a Roche LightCycler480 instrument (Roche, Basel, Switzerland). The *18S* rRNA gene was used as an internal control, and primers were used for qPCR are listed in Supplementary Table S4. QPCR was performed using SYBR Green (Toyobo) and the expression levels were calculated based on the 2^−ΔΔCT^ method. Each sample was tested with three replicates. The primers used for qPCR are listed in Supplementary Table S4.

### Plasmid construction, and expression and purification of recombinant PfILP

The cDNA encoding PfILP mature polypeptide was amplified using sequence specific primers (Supplementary Table S4) containing *EcoR* I and *Hind* III restriction sites. After double digestion with *EcoR* I and *Hind* III, the products was cloned in-frame into the *EcoR* I/*Hind* III sites of the pET-28α expression vector to generate an N-terminal polyhistidine-tagged version of PfILP lacking its signal peptide (amino acids 1–32). The recombinant plasmid were transformed into *E*. *coli Transetta* (DE3) cells which grown overnight in liquid Luria-Bertani (LB) medium supplemented with 100 mg/ml kanamycin and 100 mg/ml penicillin under constant shaking (220 rpm) at 37 °C. The cultures was then diluted 1:50 into fresh LB with penicillin and kanamycin, and grown until an OD_600_ of 0.5–0.7 under the same conditions. The bacterial was then induced with 1 mM isopropyl-β-D- thiogalactopyranoside (IPTG) and incubation continued at 37 °C or a further 12 h. The bacterial cells were pelleted by centrifugation at 8,000 × g for 5 min. Inclusion bodies were washed twice with solution I (100 mM NaCl, 50 mM Tris-HCl, 1 mM Ethylenediaminetetraacetic acid (EDTA), 1 mM Dithiothreitol (DTT), 0.5% Triton X-100, pH 8.0) and solutions II (1 mM EDTA, 50 mM Tris-HCl, 1 mM DTT, pH 8.0) for 30 min each time, respectively. Inclusion bodies were then solubilised in 50 ml of denaturing buffer comprising 100 mM Tris-HCl, 8 M urea, 1 mM glycine, 1 mM EDTA, 100 mM 2-Hydroxy-1-ethanethiol (p-ME), 10 mM DTT, 1 mM reduced glutathione (GSH), 0.1 mM oxidised glutathione (GSSG) and 10 mM imidazole (pH 9.0), and incubated at 4 °C for 24 h under constant rotation. Dissolving solution were dialysed at 4 °C and five concentrations of urea (6 M, 4 M, 2 M, 1 M and 0.5 M) in Tris-HCl buffer (20 mM Tris-HCl, 60 mM glycine, 0.5 mM DTT, 0.08 mM GSSG, 0.8 mM GSH, 5% glycerine, 6 M urea, pH 9.0) were tested. Refolded samples were centrifuged at 12,000 × g for 10 min at 4 °C and the supernatant was used for further purification. Recombinant protein was purified using a HisPurTM Ni-NTA Resin (Thermo Fisher Scientific, USA) according to the manufacturer’s protocol. The purity of eluted samples was analysed by 10% sodium dodecyl sulphate-polyacrylamide gel electrophoresis (SDS-PAGE) and stained with Coomassie Brilliant Blue R-250. Protein concentrations were determined by Modified BCA Protein Assay Kit (Sangon Biotech, Shanghai, China) following the manufacturer’s instructions.

### Primary culturing of mantle cells

Oysters were maintained in a sterile seawater tank containing 0.024 g/l ampicillin and 0.02 g/l gentamicin for two days prior to experimentation. The outside of the shell was rapidly wiped with 72% ethanol. Shells were opened and the mantle was dissociated. The primary mantle cells were cultured according to a previous study^60^.

### Cell Counting Kit-8 assays and PCNA expression analysis

Cell Counting Kit-8 assays were performed to test the effects of recombinant PfILP on mantle cell growth. Mantle cells (2000 cells/well) were isolated and seeded in 96-well plastic plates and cultured with 2.5% fetal calf serum. After culturing for 24 h, various concentrations (0, 0.25, 0.5, 1, 1.5, and 2 μg/ml) of recombinant PfILP was added and culturing continued for a further 24 h. Cell proliferation was then assayed using the Cell Counting Kit-8 following the manufacturer’s instructions. PCNA (Proliferating cell nuclear antigen, from pearl oyster) gene expression levels was measured as an indicator of cell proliferation status.

### Effects of PfILP on PfIRR and Western blot assays

Co-immunoprecipitation (Co-IP) analysis of the interaction between PfILP and PfIRR was carried out using the Pierce Classic IP Kit (#26146; Pierce, Rockford, IL, USA) according to the manufacturer’s instructions. Mantle cells were treated with 1 mM PfILP overnight at 4 °C, and Mantle cells were lysed and immunoprecipitated using a Pierce Classic IP Kit (#26146; Pierce, Rockford, IL, USA). Lysates were co-immunoprecipitated using anti-His-PfILP antibodies, subjected to SDS-PAGE (10%) and detected by western blotting with an anti-pfIRR antibody (10 μg) from our lab. Co-IP lysates obtained using anti-pfIRR antibodies (10 μg) were detected by western blotting with anti-His-PfILP (10 μg). Relative expression levels of *Pfirr* were analysed after a 24 h incubation with PfILP at various concentrations. pfIRR protein expression levels were analysed after a 1 h incubation with PfILP at various concentrations, or with 1 μg/ml PfILP for various times, by western blotting according described previously^60^. Glyceraldehyde-3-phosphate dehydrogenase (GAPDH) was used as a reference.

### PfILP stimulation in primary mantle cells

First, mantle cells (5 × 10^5^ cells/well) were seeded in 24-well plates (Corning, NY, USA). One group were incubated for 24 h with recombinant PfILP protein and RNA was subsequently extracted and subjected to qPCR analysis. Primers used for genes involved in the PfILP signalling pathway (*pfirr*, *Pfirs1*, *Pfpik3r1*, *Pfakt*, *Pfsos2*, *Pfrap-1*, *Pfraf*, *Pferk*, *GK*, *GSK-3β* and *PP1*), as well as the *18S* rRNA gene, are listed in Supplementary Table S4.

The other group of mantle cells were incubated with PfILP protein (1 μg/ml) for various time periods (0, 5, 10, 30, 60 or 240 min) then analysed by western blotting to detect the phosphorylation of Akt/PKB at residue T308, as well as the amount of Akt/PKB, phosphorylated p44/42 MAPK, p44/42 MAPK, GSK-3β, PP1 and GAPDH. Mantle cells were incubated with PfILP at different concentrations (0, 0.25, 0.5, 0.75, 1.0 or 2.0 μg/ml) for 30 min. Lysates were subjected to western blotting with appropriate antibodies, and the densitometry ratios of phospho-p44/42/total p44/42, phospho-Akt/total Akt, GSK-3β/GAPDH, and PP1/GAPDH were measured using Lane 1D software from Minichemi 610.

### Cell cycle analysis

Because mantle primary cells display weak cell proliferation, we used human 293 T cells instead, and these cells were cultured in Dulbecco’s Modified Eagle Medium (DMEM) supplemented with 10% fetal bovine serum at 37 °C with 5% CO_2_. After 3 days, 293 T cells (5 × 10^4^ cells/well) were seeded in 24-well plates (Falcon, Corning, NY, USA). When cells had grown to 80%, the medium was discarded, and different concentrations of recombinant PfILP protein were added with 2% serum medium and cultured at 37 °C with 5% CO_2_ for 36 h. Phosphate-buffered saline (PBS) was used instead of recombinant PfILP protein in the negative control group. Samples were collected and washed twice with PBS, resuspended in 1.5 ml of pre-cooled 80% ethanol, mixed, and incubated overnight at 4 °C in the dark. Samples were collected and washed twice with PBS, then mixed with 0.5 ml FxCycle PI/RNase Staining Solution (Thermo Fisher Scientific) and incubated at room temperature for 30 min in the dark. Samples were collected and filtered through a 40 μm cell strainer and analysed by FACScan flow cytometry (Philadelphia, Pennsylvania, USA) at 488 nm.

### Statistical analysis

All data graph were analysed by GraphPad Prism 6 (GraphPad Software, La Jolla, CA, USA). Values were displayed the means ± SEM and analyzed by Student’s t-test. SPSS software (version 18.0) (SPSS, Chicago, IL, USA) was used for the statistical analysis. A *p*-value < 0.05 or <0.01 was considered statistically significant.

## Supplementary information

Supplementary Information.

## Data Availability

The datasets used and/or analyzed during the current study are available from the corresponding author on reasonable request.
