# Supplementary material for: The role of a new insulin-like peptide in the pearl oyster Pinctada fucata martensii
Source: Sci Rep. 2020 Jan 16;10:433. doi: 10.1038/s41598-019-57329-3 (PMC6965660; doi:10.1038/s41598-019-57329-3)

The role of a new insulin-like peptide in the pearl oyster *Pinctada fucata martensii*

Hua Zhang[1,2], Maoxian He[1,2*]

[1] *CAS Key Laboratory of Tropical Marine Bio-resources and Ecology, South China Sea Institute of Oceanology, Chinese Academy of Sciences, Guangzhou 510301, China*

[2] *Guangdong Provincial Key Laboratory of Applied Marine Biology, Guangzhou 510301, China*

[*]**Corresponding author**:

Dr. Maoxian He, CAS Key Laboratory of Tropical Marine Bio-resources and Ecology, South China Sea Institute of Oceanology, Chinese Academy of Sciences, Guangzhou 510301, China

Tel: +86-20-89023144, Fax: +86-20-84458964, E-mail Address: hmx2@scsio.ac.cn

Supplementary information

Table S1 Description of ILP signaling pathway cDNAs in *P. fucata martensii*

Table S2 Species name, protein name and GenBank No from species were used in Supplementary Fig. 7

Table S3 Species name, protein name and GenBank No were used in Fig.1D

Table S4 Primers used for cloning and expression analysis in this study

Supplementary Figure. 1 A: The structural domain of PfIRS1 protein；B: Comparison of PfIRS1 amino acid sequences with its orthologs from others species; C: The phylogenic tree (Maximum likelihood) of PfIRS1 protein was constructed using MEGA 6.0.

Supplementary Figure. 2 A: The structural domain of PfPIK3R1 protein；B: Comparison of PfPIK3R1 amino acid sequences with its orthologs from others species; C: The phylogenic tree (Maximum likelihood) of PfPIK3R1 protein was constructed using MEGA 6.0.

Supplementary Figure. 3 A: The structural domain of PfAKT protein；B: Comparison of PfAKT amino acid sequences with its orthologs from others species; C: The phylogenic tree (Maximum likelihood) of PfAKT protein was constructed using MEGA 6.0.

Supplementary Figure. 4 A: The structural domain of PfSOS2 protein；B: Comparison of

PfSOS2 amino acid sequences with its orthologs from others species; C: The phylogenic tree (Maximum likelihood) of PfSOS2 protein was constructed using MEGA 6.0.

Supplementary Figure. 5 A: The structural domain of PfRap-1 protein；B: Comparison of PfRap-1 amino acid sequences with its orthologs from others species; C: The phylogenic tree (Maximum likelihood) of PfRap-1 protein was constructed using MEGA 6.0.

Supplementary Figure. 6 A: The structural domain of PfRaf protein；B: Comparison of PfRaf amino acid sequences with its orthologs from others species; C: The phylogenic tree (Maximum likelihood) of PfRaf protein was constructed using MEGA 6.0.

Supplementary Figure. 7 Multiple sequence alignment of the amino acid sequence of PfILP with various insulin superfamily proteins. The six conserved cysteines are marked with a black box.

Table S1

Gene	GenBank acc. No	Amplicon size (bp)	ORF (aa)	BLASTp best hit (Reference/species)	Identity (%)
PfILP	MK064538	1443	170	XP_011455161.1/ *Crassostrea gigas*	31.32
Pfirs1	MK064539	5388	1146	XP_022311786.1/*Crassostrea virginica*	50.17
Pfpik3r1	MK064540	5848	942	EKC33889.1/*Crassostrea gigas*	68.87
Pfakt	MK064541	4012	487	ALN96976.1/*Crassostrea hongkongensis*	86.84
Pfsos2	MK064542	6984	1268	XP_011455479.1/*Crassostrea gigas*	70.08
Pfrap-1	MK064543.	2156	185	XP_011455539.1/*Crassostrea* gigas	89.19
Pfraf	MK064544	3916	703	XP_022299579.1/*Crassostrea virginica*	67.22

Table S2

Species name	Protein name	GenBank No.
Crassostrea gigas	ILP	XP_011455161.1
Crassostrea gigas	Insluin	EKC18433.1
Callorhinchus milii	IGF1	AFK11290.1
Drosophila melanogaster	ILP1	AAF50205.1
Sinonovacula consricta	ILP1	KR534869.1
Sinonovacula consricta	ILP2	KR534870.1
Mizuhopecten yessoensis	ILP precursor	BAD13420.1
Homo sapiens	insulin	AAA59172.1
Homo sapiens	Relaxin	CAA00599.1
Homo sapiens	IGF1	CAG46659.1
Homo sapiens	IGF2	AAA60088.1
Danio rerio	Insulin	CAC20109.1
Danio rerio	Relaxin	AEL22115.1
Danio rerio	IGF1	AAI14263.1
Danio rerio	IGF2a	AAH85623.1

Table S3

**Species name**	**Protein name**	**GenBank No.**
Homo sapiens	Relaxin 1	P04808.1
Homo sapiens	Relaxin 2	P04090.1
Homo sapiens	Relaxin 3	Q8WXF3.1
Homo sapiens	ILP 3	P51460.2
Homo sapiens	ILP 5	Q9Y5Q6.2
Homo sapiens	insulin	AAA59172.1
Homo sapiens	IGF 1	CAG46659.1
Homo sapiens	IGF 2	AAA60088.1
Mus musculus	Relaxin 2	CAA81611.1
Mus musculus	Relaxin 3	Q8CHK2.1
Mus musculus	ILP 3	O09107.2
Mus musculus	ILP 6	Q9QY05.1
Danio rerio	insulin	CAC20109.1
Danio rerio	Relaxin	AEL22115.1
Danio rerio	IGF 1	AAI14263.1
Danio rerio	IGF 2a	AAH85623.1
Cherax destructor	IAG	ACD91988.1
Cherax quadricarinatus	IAG	ABH07705.1
Penaeus japonicus	IAG	BAK20460.1
Penaeus monodon	IAG	ADA67878.1
Penaeus chinensis	IAG 2	AFU60549.1
Sagmariasus verreauxi	IAG	AHY99679.1
Crassostrea virginica	IGF 1	XP_022295716.1
Crassostrea virginica	ILP 3	XP_022291565.1
Crassostrea gigas	ILP	XP_011455161.1
Crassostrea gigas	Insulin	EKC18433.1
Crassostrea gigas	ILP 7	EKC19125.1
Callorhinchus milii	IGF 1	AFK11290.1
Sinonovacula consricta	ILP 1	KR534869.1
Sinonovacula consricta	ILP 2	KR534870.1
Mizuhopecten yessoensis	ILP	BAD13420.1
Centruroides sculpturatus	ILP	XP_023214050.1

Table S4

Primers	sequence（5'-3'）
**Race**	
UPM long	CTAATACGACTCACTATAGGGCAAGCAG
	TGGTATCAACGCAGAGT
UPM short	CTAATACGACTCACTATAGGGC
NUP	AAGCAGTGGTATCAACGCAGAGT
5RCPfILP3	ATTTGTGCTTGTTTTCTCGTTCTCA
5RCPfILP2	CAGCATTGTATGGAAAACCTGAGTC
5RCPfILP1	GTTGTTGTTTGTGGAGATTGTGCTG
3racePfRap-1 1774-58	TCAATAATGCTAAAGGCAATGTG
3racePfRap-1 1876-57.4	CGTATTTATTCTTTCTCGGCAA
3racePfRap-1 1933-58.2	ATGCTTTTTCTCTGAACTTGGG
3racePfRap-1 1985-57.5	CATGGTTTGTATGCAGTAGTGAGA
5racePfRap-1 167-58.9	TACTCCCTCATTTTGCTTCCTG
5racePfRap-1 206-57.7	CTTTTTCCTACACCACCACTACC
5racePfRap-1 519-58	CTCTAAATCACACTTGTTTCCGAC
5racePfRap-1 423-58.2	TGTTGACTGGGCTGTGATAGAA
3racePfRaf828-60.8	GAGCAGCGTCCTTTATTTCCAC
3racePfRaf906-56.8	TCGTCAGAACCAACTCTCAATC
3racePfRaf1147-59.8	TTATATTCCGTGTACTTGCCCG
3racePfRaf1064-57.1	GGAGTTGTTTACATAACGGCAG
5racePfRaf119-58.8	TGATCCTATCCTTTCGTTCACAG
5racePfRaf215-58	TGCTTTAAGCTGTTGTGGTGTT
5racePfRaf261-56.4	TATTTGTATGCCTTGTTTTCCTC
5racePfRaf404-60.3	CGTTTGCCTTGCGATTTCTATA
3racePfSos2380-58.5	AATCACACACCAGAGTCAGCCT
3racePfSos2551-57.8	TCCACTGCCTAGACCTACGC

3racePfSos2580-61.3	TACAGCCCCCACCATTACCTC
3racePfSos2772-59.3	GACTCGTTTCATAGTGGACAACTTC
5racePfSos136-58.8	GAATAGTGTAAGCACAACGAGCC
5racePfSos267-58.8	AAATCGAAGCAGTTCAGCAATT
5racePfSos393-59.3	AGGAGGTTCAGGATCAGACCC
5racePfSos496-57.1	GCATCTCCCATAGCCCACT
3racePfAKT721-58	TAATGGAGTATGTGAATGGAGGAG
3racePfAKT935-56	AGAGATGTACTACGGAGCAAGC
3racePfAKT1069-58.2	TGTGTGGACGATTACCGTTCTA
3racePfAKT1251-58.6	GCACATCCATTCTTCAAATCAAT
5racePfAKT299-61.8	TGGCTTCTCCTGCTTCATCAAC
5racePfAKT344-58.6	CACCACCGTCCATTGTAAGC
5racePfAKT218-58.6	CCTGTAACCTAGAAAGGAACCATC
5racePfAKT397-60.3	GCTATCCAATCTTCCCGTTCC
3race PfPIK3R1 1775-58	TAAGCATCCAGGAAAGTCGC
3race PfPIK3R1 1987-59.7	CCCCACAATAACAAGAGCCTG
3race PfPIK3R1 2090-58.	AGAACGATCCTACCAGCGAATA
3race PfPIK3R1 2276-57.2	ATGTACGCATGTTGTACCCTGT
5race PfPIK3R1 132-57.6	TTCACAGCGTCTGGCTACTAAC
5race PfPIK3R1 239-58.4	GCCGACTGAACTAATAGGATGG
5race PfPIK3R1 459-60.8	TGTAGCATAGGGCCTCGAATCT
5race PfPIK3R1 712-57.6	AAGGTTTACTTGTGCTGGGTCT
3racePfIRS873-58.3	AATAGGGAGCCCACACCAGT
3racePfIRS903-59.7	CAGTATTGTGAGTCCTGTCGGC
3racePfIRS978-58.2	AGATATGTACGGCTCAAGTCCAG
3racePfIRS1116-59	CTCCAGGGAAAAGTCTGAACG
5racePfIRS118-60.3	CGTTGTCATAGTATTCCAGTCTTGC
5racePfIRS277-58.9	ATGACATCTCGTCTTCCTCGG
5racePfIRS413-57.3	GTCCCGTGTTGAATCTTGTGT

5racePfIRS601-60	TGGCAGCATCGTCTACTTTCAT
**QPCR**	
dR: Pfsos:56.6-2938	CTTGTATGGTGATGAGTCCGAT
dF: Pfsos:57.3-46	TTGGTATGGCCTTAGATTAAATTAG
dF: Pferk262-59.9	TTAGGGGCACCGACAGTAGA
dR: Pferk353-60	TGCTGGGTCTTAAGGAGCTTG
dF: Pfirs473-59.97	GGACGTATCGTCTCTGCCTG
dR: Pfirs619-59.97	GCGCTGATCTTCCTACCTCC
dF: Pfraf306-60	ACCTGACATTCCTCAGCGTG
dR: Pfraf434-59.4	TTCCCAGGTTCTATCCGCAC
dF: PfRap-1575-60	TGCTGGAGCCATCCCTATCT
dR: PfRap-1661-59.7	TGCCTGAGATGTCGACACAG
dF: Pfakt237-60	ATGGACGGTGGTGGTAGAGA
dR: Pfakt330-60	CAGATCTTCGGCTACGGACC
18sF	CGTTTCAACAAGACGCCAGTAG
18sR	ACGAAAAAAAGGTTTGAGAGACG
d PfILP-F	TTCCATGGTCCCGGAAAGAG
dPfILP-R	AGCCTCAATATCCGGTGCCT
Pfirr-F	AGACGGAGACGGGAAAGAAG
Pfirr-R	CCCCCAACAGACGTACAACA
GK-F	ATTCCGACTCCCTTGGTATG
GK-R	GACGGGTGATTTGTCTTTTT
GSK-3β-F	CAACCACCCGACTTCCTAAC
GSK-3β-R	GGATGCCCTTCATTCCCAGC
PP1-F	TGGTGAAGACAAAAGTGGAT
PP1-R	TGGTCATATGTTGAGAGGTG
PCNA-F	CACACGTTGCTTTGGTAGCG
PCNA-R	ATCATCGTTGCTGGCACACT
**Clone**	

EcoR I(sense)：	5'-CGGAATTCAACATACAGGCATGTGGTT CTGATC
Hind III(antisense)：	5'-CCCAAGCTTTTTCCTATTGCCCATACGT CGTATC

Supplementary Figure. 1

A

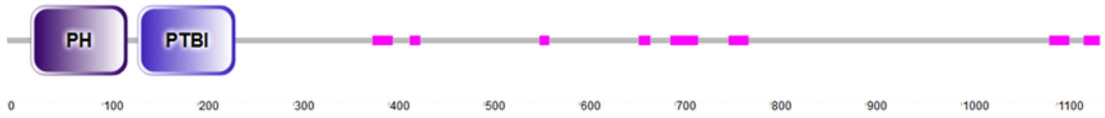

B

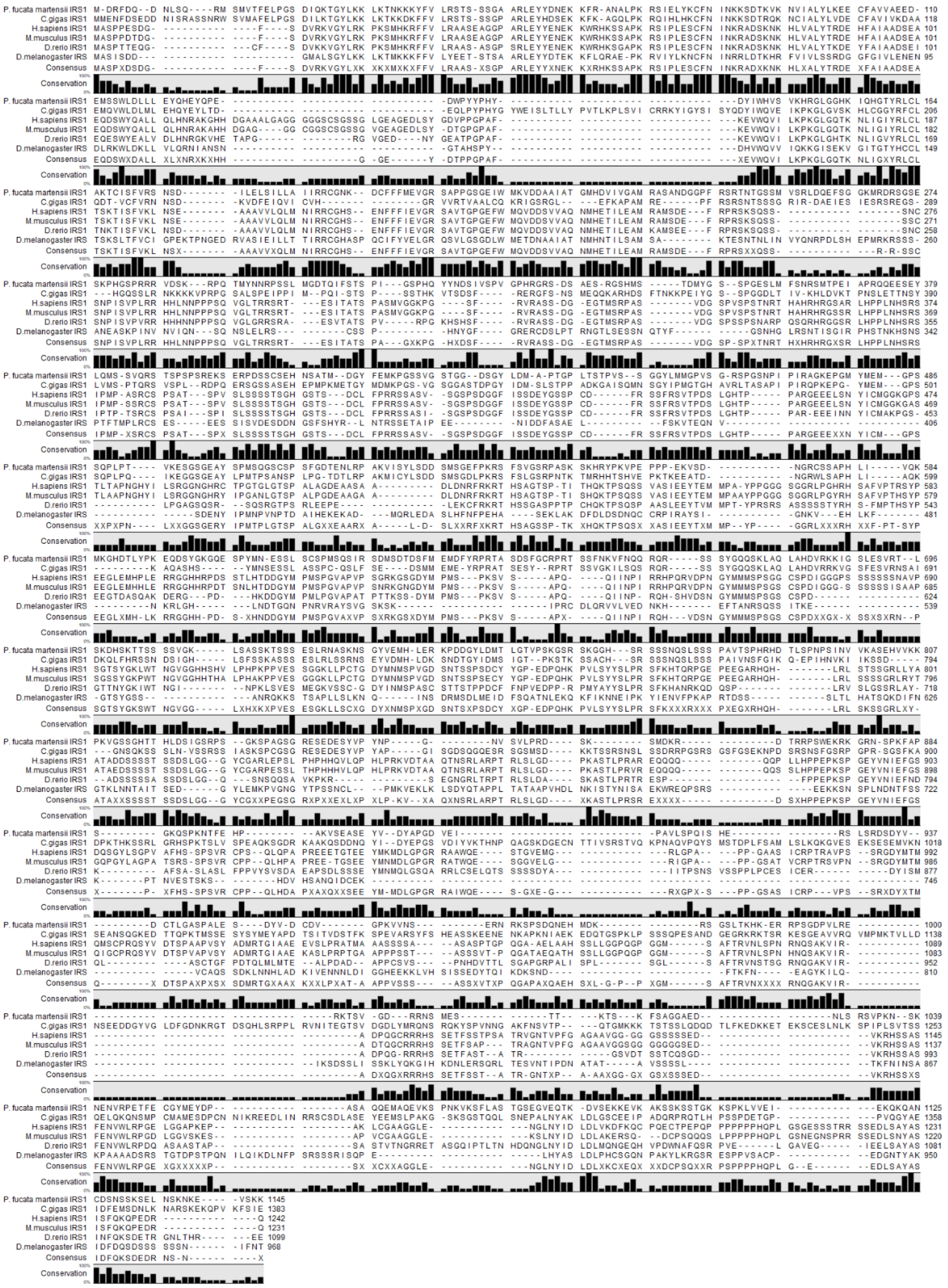

C

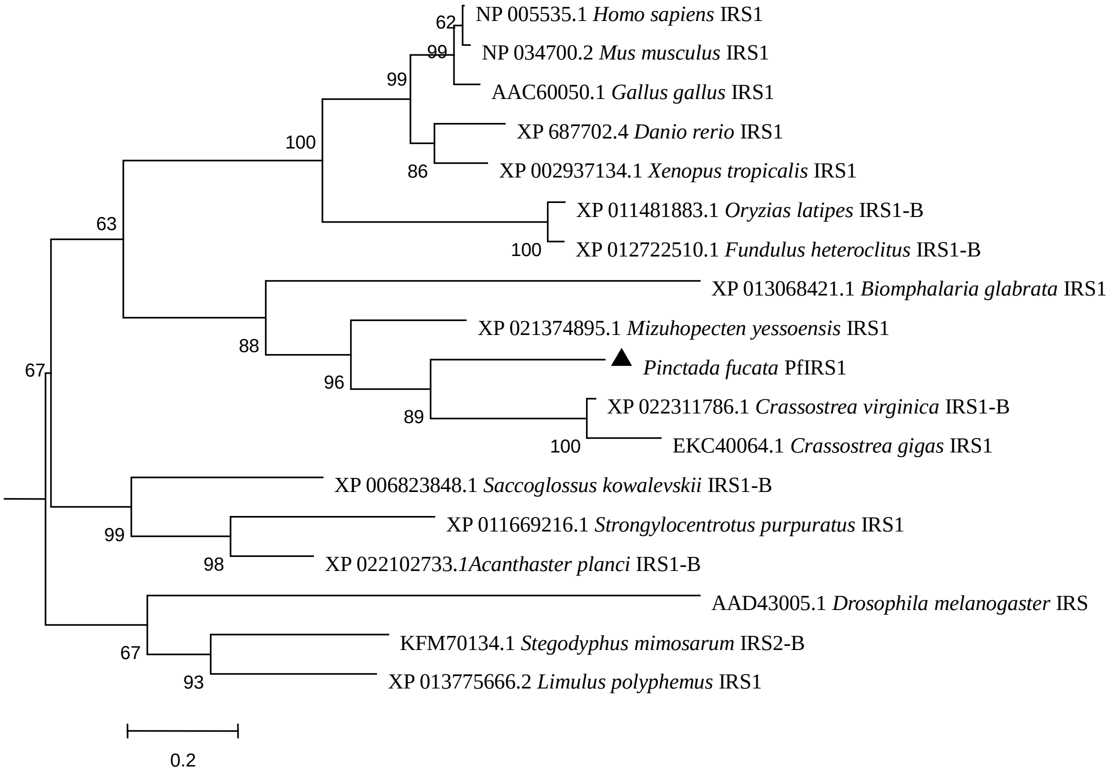

Supplementary Figure. 2

A

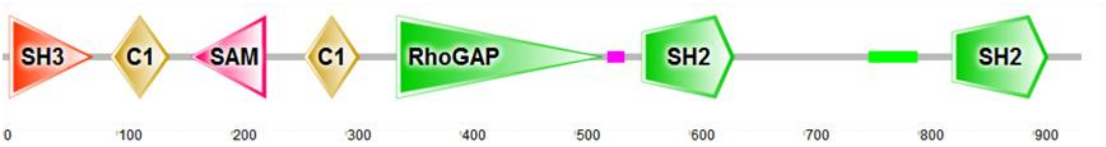
SH3
C1
SAM
C1
RhoGAP
SH2
SH2
0
100
200
300
400
500
600
700
800
900

B

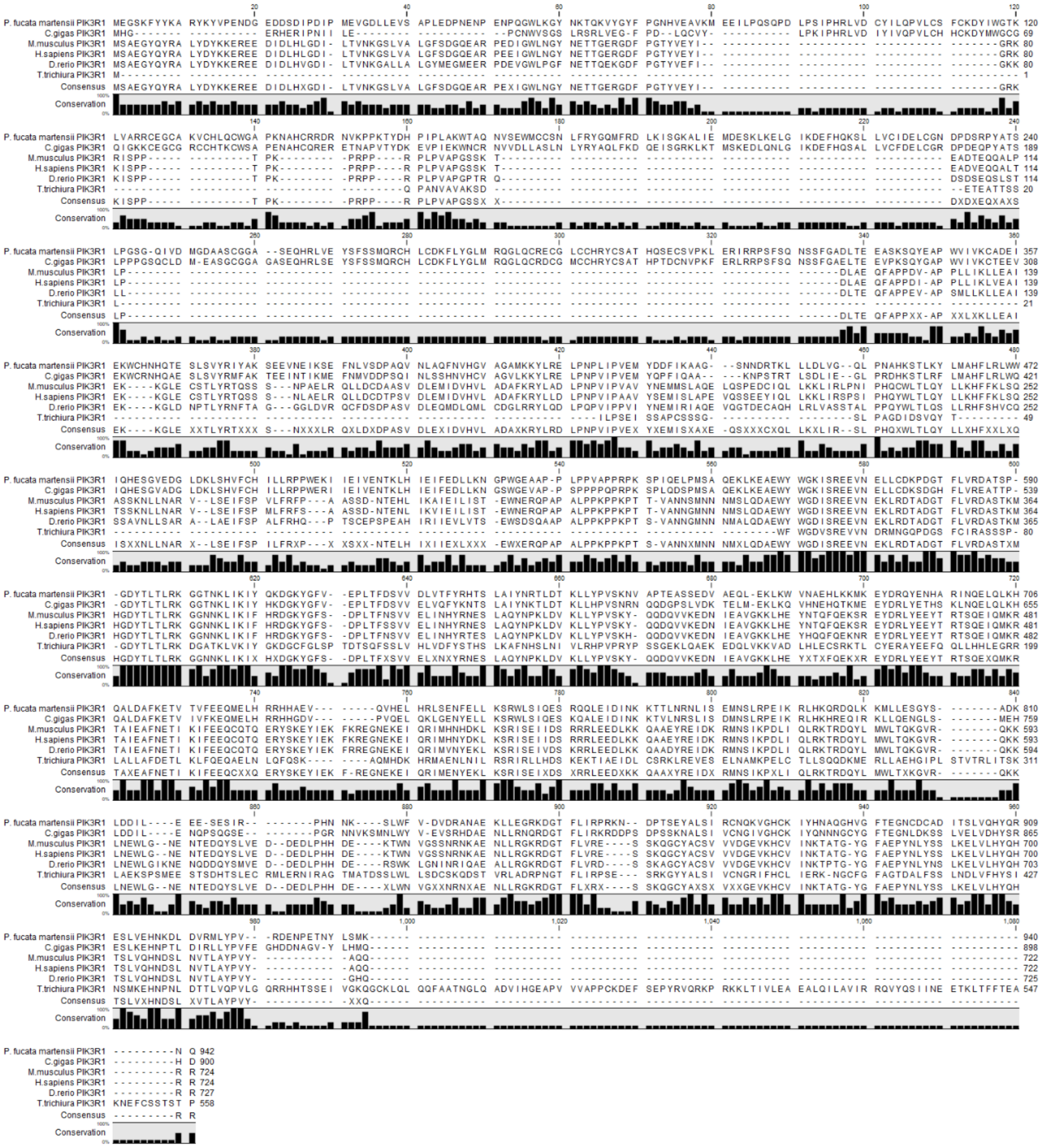
P. fucata martensii PIK3R1
C.gigas PIK3R1
M.musculus PIK3R1
H.sapiens PIK3R1
D.rerio PIK3R1
T.trichiura PIK3R1
Consensus
Conservation

C

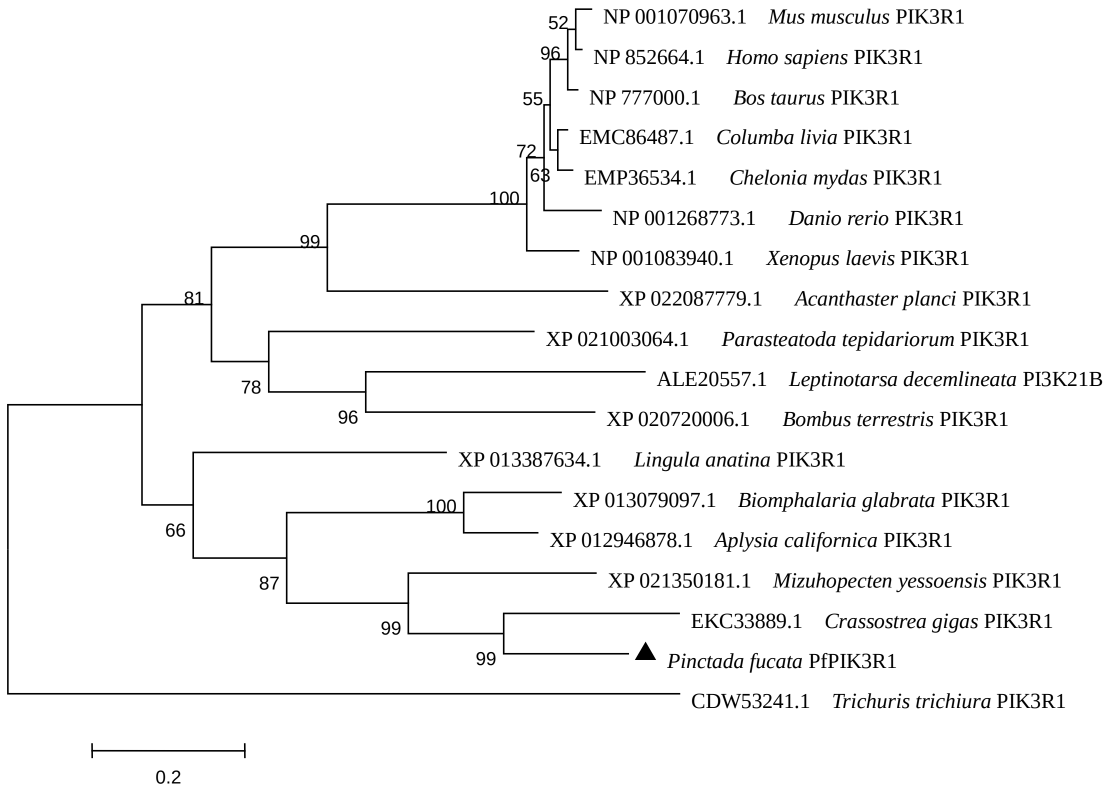

Supplementary Figure. 3

A

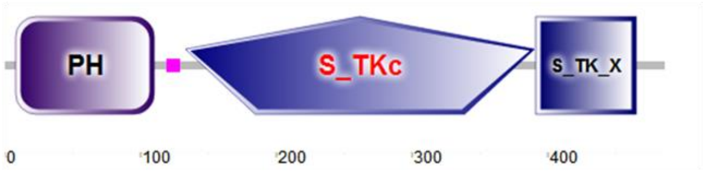

PH
S_TKc
S_TK_X
0
100
200
300
400

B

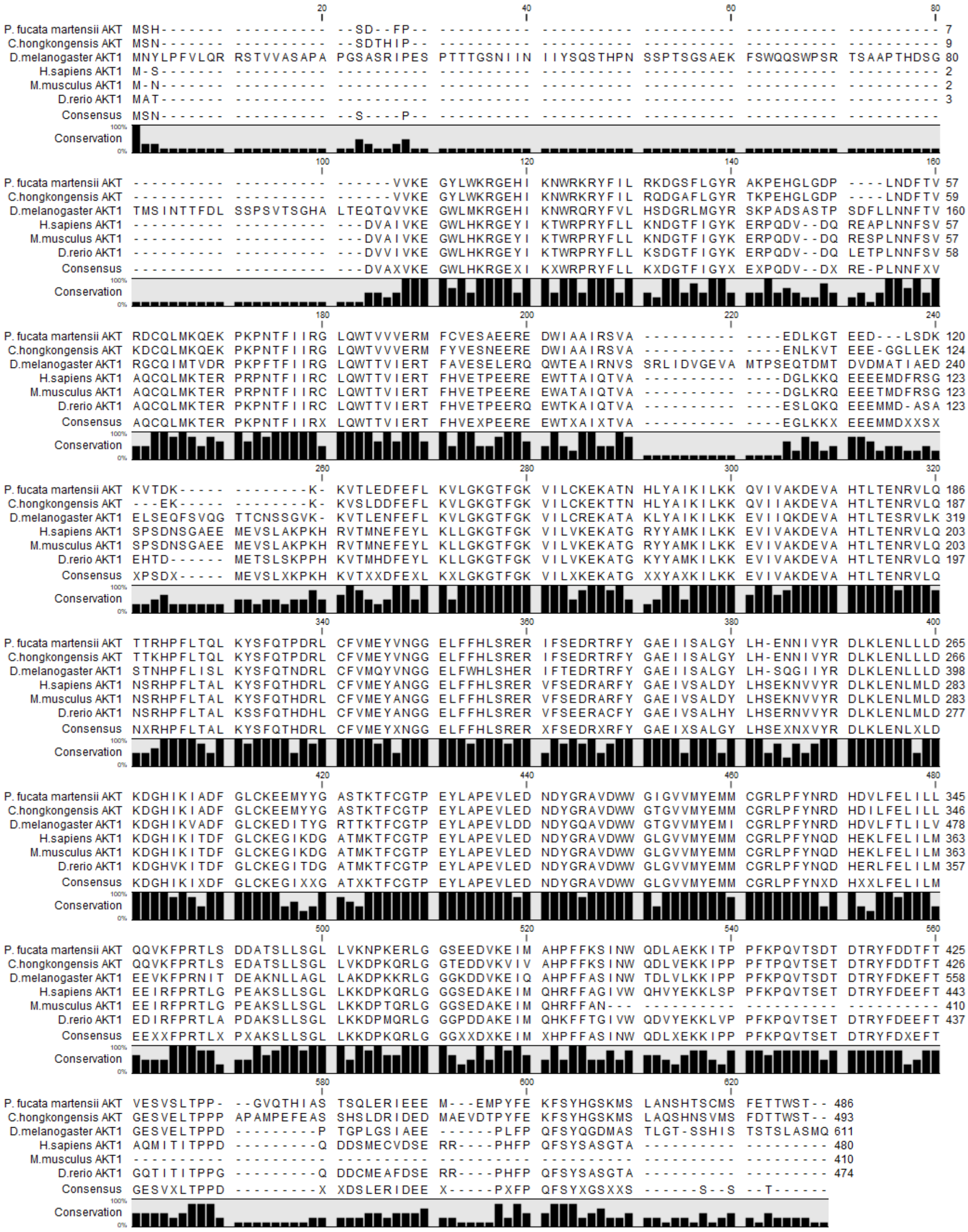

20 40 60 80
P. fucata martensii AKT MSH------- ---------- --SD--FP-- ---------- ---------- ---------- ---------- ---------- 7
C.hongkongensis AKT MSN------- ---------- --SDTHIP-- ---------- ---------- ---------- ---------- ---------- 9
D.melanogaster AKT1 MNYLPFVLQR RSTVVASAPA PGSASRIPES PTTTGSNIIN IIYSQSTHPN SSPTSGSAEK FSWQQSWPSR TSAAPTHDSG 80
H.sapiens AKT1 M-S------- ---------- ---------- ---------- ---------- ---------- ---------- ---------- 2
M.musculus AKT1 M-N------- ---------- ---------- ---------- ---------- ---------- ---------- ---------- 2
D.rerio AKT1 MAT------- ---------- ---------- ---------- ---------- ---------- ---------- ---------- 3
Consensus MSN------- ---------- --S----P-- ---------- ---------- ---------- ---------- ----------
Conservation
100 120 140 160
P. fucata martensii AKT ---------- ---------- ------VVKE GYLWKRGEHI KNWRKRYFIL RKDGSFLGYR AKPEHGLGDP ----LNDFTV 57
C.hongkongensis AKT ---------- ---------- ------VVKE GYLWKRGEHI KNWRKRYFIL RQDGAFLGYR TKPEHGLGDP ----LNDFTV 59
D.melanogaster AKT1 TMSINTTFDL SSPSVTSGHA LTEQTQVVKE GWLMKRGEHI KNWRQRYFVL HSDGRLMGYR SKPADSASTP SDFLLNNFTV 160
H.sapiens AKT1 ---------- ---------- ---DVAIVKE GWLHKRGEYI KTWRPRYFLL KNDGTFIGYK ERPQDV--DQ REAPLNNFSV 57
M.musculus AKT1 ---------- ---------- ---DVAIVKE GWLHKRGEYI KTWRPRYFLL KNDGTFIGYK ERPQDV--DQ RESPLNNFSV 57
D.rerio AKT1 ---------- ---------- ---DVVIVKE GWLHKRGEYI KTWRPRYFLL KSDGTFIGYK ERPQDV--DQ LETPLNNFSV 58
Consensus ---------- ---------- ---DVAXVKE GWLHKRGEXI KXWRPRYFLL KXDGTFIGYX EXPQDV--DX RE-PLNNFXV
Conservation
180 200 220 240
P. fucata martensii AKT RDCQLMKQEK PKPNTFIIRG LQWTVVVERM FCVESAEERE DWIAAIRSVA ---------- ----EDLKGT EED---LSDK 120
C.hongkongensis AKT KDCQLMKQEK PKPNTFIIRG LQWTVVVERM FYVESNEERE DWIAAIRSVA ---------- ----ENLKVT EEE-GGLLEK 124
D.melanogaster AKT1 RGCQIMTVDR PKPFTFIIRG LQWTTVIERT FAVESELERQ QWTEAIRNVS SRLIDVGEVA MTPSEQTDMT DVDMATIAED 240
H.sapiens AKT1 AQCQLMKTER PRPNTFIIRC LQWTTVIERT FHVETPEERE EWTTAIQTVA ---------- ----DGLKKQ EEEEMDFRSG 123
M.musculus AKT1 AQCQLMKTER PRPNTFIIRC LQWTTVIERT FHVETPEERE EWATAIQTVA ---------- ----DGLKRQ EEETMDFRSG 123
D.rerio AKT1 AQCQLMKTER PKPNTFIIRC LQWTTVIERT FHVETPEERQ EWTKAIQTVA ---------- ----ESLQKQ EEEMMD-ASA 123
Consensus AQCQLMKTER PKPNTFIIRX LQWTTVIERT FHVEXPEERE EWTXAIXTVA ---------- ----EGLKKX EEEMMDXXSX
Conservation
260 280 300 320
P. fucata martensii AKT KVTDK----- --------K- KVTLEDFEFL KVLGKGTFGK VILCKEKATN HLYAIKILKK QVIVAKDEVA HTLTENRVLQ 186
C.hongkongensis AKT ---EK----- --------K- KVSLDDFEFL KVLGKGTFGK VILCKEKTTN HLYAIKILKK QVIIAKDEVA HTLTENRVLQ 187
D.melanogaster AKT1 ELSEQFSVQG TTCNSSGVK- KVTLENFEFL KVLGKGTFGK VILCREKATA KLYAIKILKK EVIIQKDEVA HTLTESRVLK 319
H.sapiens AKT1 SPSDNSGAEE MEVSLAKPKH RVTMNEFEYL KLLGKGTFGK VILVKEKATG RYYAMKILKK EVIVAKDEVA HTLTENRVLQ 203
M.musculus AKT1 SPSDNSGAEE MEVSLAKPKH RVTMNEFEYL KLLGKGTFGK VILVKEKATG RYYAMKILKK EVIVAKDEVA HTLTENRVLQ 203
D.rerio AKT1 EHTD------ METSLSKPPH KVTMHDFEYL KLLGKGTFGK VILVKEKATG KYYAMKILKK EVIVAKDEVA HTLTENRVLQ 197
Consensus XPSDX----- MEVSLXKPKH KVTXXDFEXL KXLGKGTFGK VILXKEKATG XXYAXKILKK EVIVAKDEVA HTLTENRVLQ
Conservation
340 360 380 400
P. fucata martensii AKT TTRHPFLTQL KYSFQTPDRL CFVMEYVNGG ELFFHLSRER IFSEDRTRFY GAEIISALGY LH-ENNIVYR DLKLENLLLD 265
C.hongkongensis AKT TTKHPFLTQL KYSFQTPDRL CFVMEYVNGG ELFFHLSRER IFSEDRTRFY GAEIISALGY LH-ENNIVYR DLKLENLLLD 266
D.melanogaster AKT1 STNHPFLISL KYSFQTNDRL CFVMQYVNGG ELFWHLSHER IFTEDRTRFY GAEIISALGY LH-SQGIIYR DLKLENLLLD 398
H.sapiens AKT1 NSRHPFLTAL KYSFQTHDRL CFVMEYANGG ELFFHLSRER VFSEDRARFY GAEIVSALDY LHSEKNVVYR DLKLENLMLD 283
M.musculus AKT1 NSRHPFLTAL KYSFQTHDRL CFVMEYANGG ELFFHLSRER VFSEDRARFY GAEIVSALDY LHSEKNVVYR DLKLENLMLD 283
D.rerio AKT1 NSRHPFLTAL KSSFQTHDHL CFVMEYANGG ELFFHLSRER VFSEERACFY GAEIVSALHY LHSERNVVYR DLKLENLMLD 277
Consensus NXRHPFLTAL KYSFQTHDRL CFVMEYXNGG ELFFHLSRER XFSEDRXRFY GAEIXSALGY LHSEXNXVYR DLKLENLXLD
Conservation
420 440 460 480
P. fucata martensii AKT KDGHIKIADF GLCKEEMYYG ASTKTFCGTP EYLAPEVLED NDYGRAVDWW GIGVVMYEMM CGRLPFYNRD HDVLFELILL 345
C.hongkongensis AKT KDGHIKIADF GLCKEEMYYG ASTKTFCGTP EYLAPEVLED NDYGRAVDWW GTGVVMYEMM CGRLPFYNRD HDILFELILL 346
D.melanogaster AKT1 KDGHIKVADF GLCKEDITYG RTTKTFCGTP EYLAPEVLDD NDYGQAVDWW GTGVVMYEMI CGRLPFYNRD HDVLFTLILV 478
H.sapiens AKT1 KDGHIKITDF GLCKEGIKDG ATMKTFCGTP EYLAPEVLED NDYGRAVDWW GLGVVMYEMM CGRLPFYNQD HEKLFELILM 363
M.musculus AKT1 KDGHIKITDF GLCKEGIKDG ATMKTFCGTP EYLAPEVLED NDYGRAVDWW GLGVVMYEMM CGRLPFYNQD HEKLFELILM 363
D.rerio AKT1 KDGHVKITDF GLCKEGITDG ATMKTFCGTP EYLAPEVLED NDYGRAVDWW GLGVVMYEMM CGRLPFYNQD HERLFELILM 357
Consensus KDGHIKIXDF GLCKEGIXXG ATXKTFCGTP EYLAPEVLED NDYGRAVDWW GLGVVMYEMM CGRLPFYNXD HXXLFELILM
Conservation
500 520 540 560
P. fucata martensii AKT QQVKFPRTLS DDATSLLSGL LVKNPKERLG GSEEDVKEIM AHPFFKSINW QDLAEKKITP PFKPQVTSDT DTRYFDDTFT 425
C.hongkongensis AKT QQVKFPRTLS EDATSLLSGL LVKDPKQRLG GTEDDVKVIV AHPFFKSINW QDLVEKKIPP PFTPQVTSET DTRYFDDTFT 426
D.melanogaster AKT1 EEVKFPRNIT DEAKNLLAGL LAKDPKKRLG GGKDDVKEIQ AHPFFASINW TDLVLKKIPP PFKPQVTSDT DTRYFDKEFT 558
H.sapiens AKT1 EEIRFPRTLG PEAKSLLSGL LKKDPKQRLG GGSEDAKEIM QHRFFAGIVW QHVYEKKLSP PFKPQVTSET DTRYFDEEFT 443
M.musculus AKT1 EEIRFPRTLG PEAKSLLSGL LKKDPTQRLG GGSEDAKEIM QHRFFAN--- ---------- ---------- ---------- 410
D.rerio AKT1 EDIRFPRTLA PDAKSLLSGL LKKDPMQRLG GGPDDAKEIM QHKFFTGIVW QDVYEKKLVP PFKPQVTSET DTRYFDEEFT 437
Consensus EEXXFPRTLX PXAKSLLSGL LKKDPKQRLG GGXXDXKEIM XHPFFASINW QDLXEKKIPP PFKPQVTSET DTRYFDXEFT
Conservation
580 600 620
P. fucata martensii AKT VESVSLTPP- --GVQTHIAS TSQLERIEEE M---EMPYFE KFSYHGSKMS LANSHTSCMS FETTWST-- 486
C.hongkongensis AKT GESVELTPPP APAMPEFEAS SHSLDRIDED MAEVDTPYFE KFSYHGSKMS LAQSHNSVMS FDTTWST-- 493
D.melanogaster AKT1 GESVELTPPD ---------P TGPLGSIAEE ------PLFP QFSYQGDMAS TLGT-SSHIS TSTSLASMQ 611
H.sapiens AKT1 AQMITITPPD ---------Q DDSMECVDSE RR----PHFP QFSYSASGTA ---------- --------- 480
M.musculus AKT1 ---------- ---------- ---------- ---------- ---------- ---------- --------- 410
D.rerio AKT1 GQTITITPPG ---------Q DDCMEAFDSE RR----PHFP QFSYSASGTA ---------- --------- 474
Consensus GESVXLTPPD ---------X XDSLERIDEE X-----PXFP QFSYXGSXXS ------S--S --T------
Conservation

C

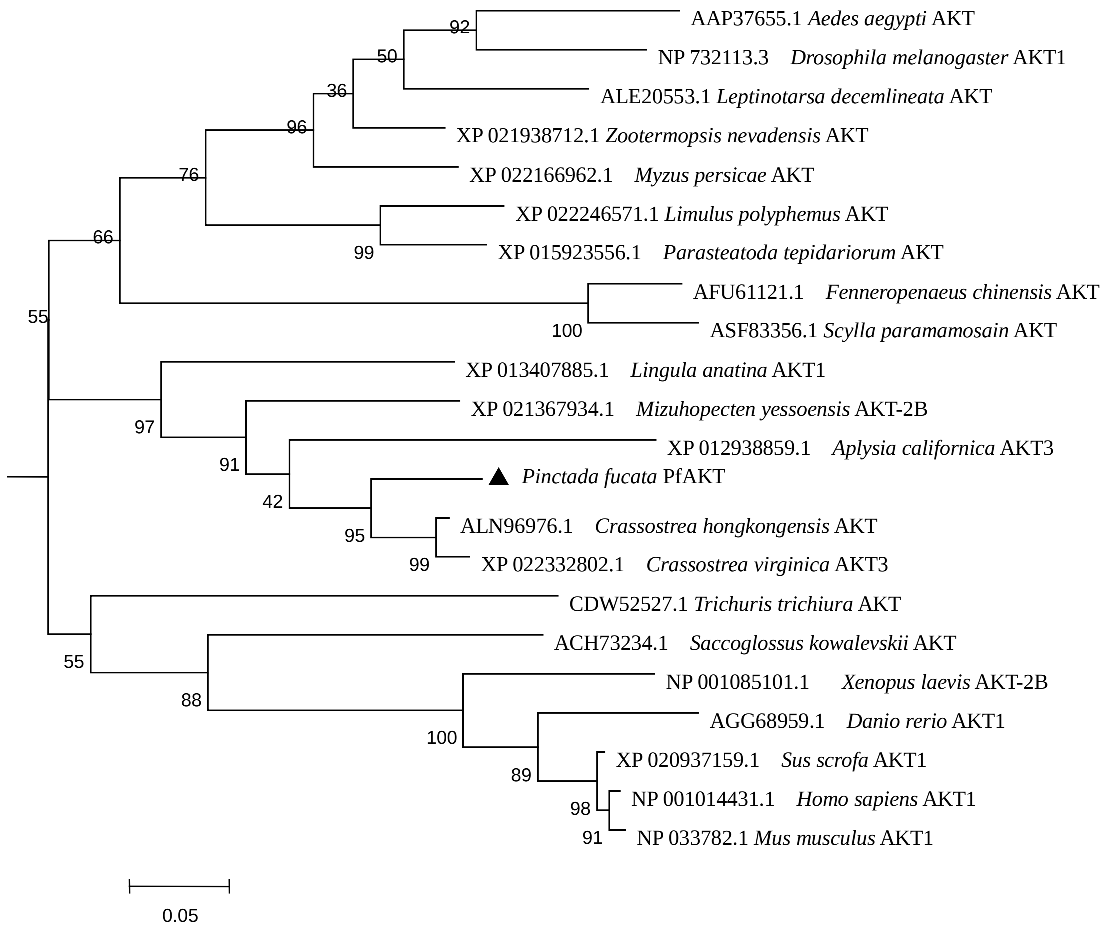

Supplementary Figure. 4

A

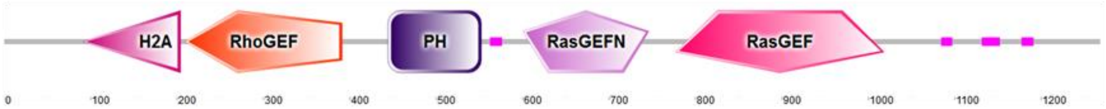

H2A
RhoGEF
PH
RasGEFN
RasGEF
0
100
200
300
400
500
600
700
800
900
1000
1100
1200

B

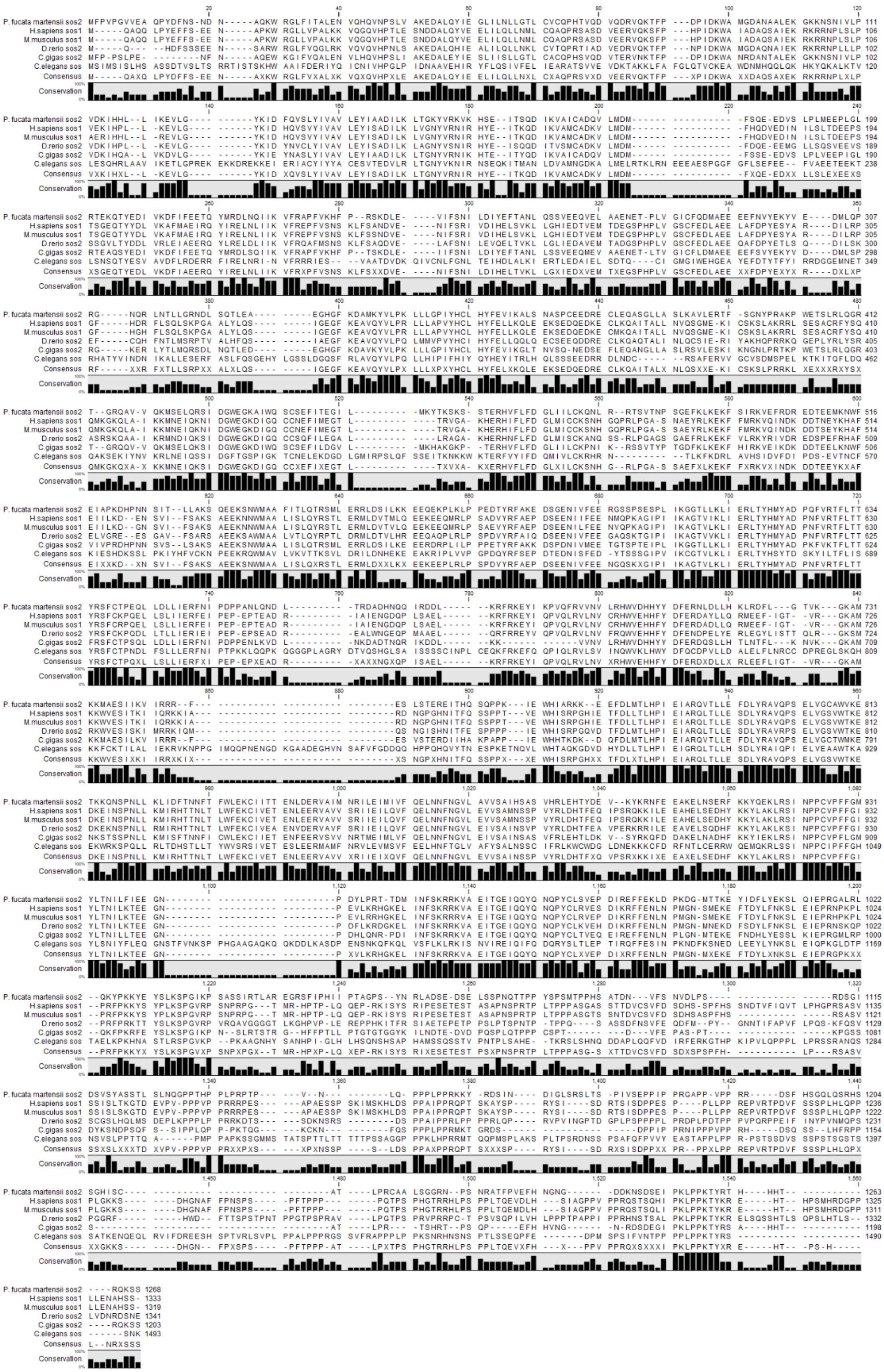
P. fucata martensii sos2
H.sapiens sos1
M.musculus sos1
D.rerio sos2
C.gigas sos2
C.elegans sos
Consensus
Conservation

C

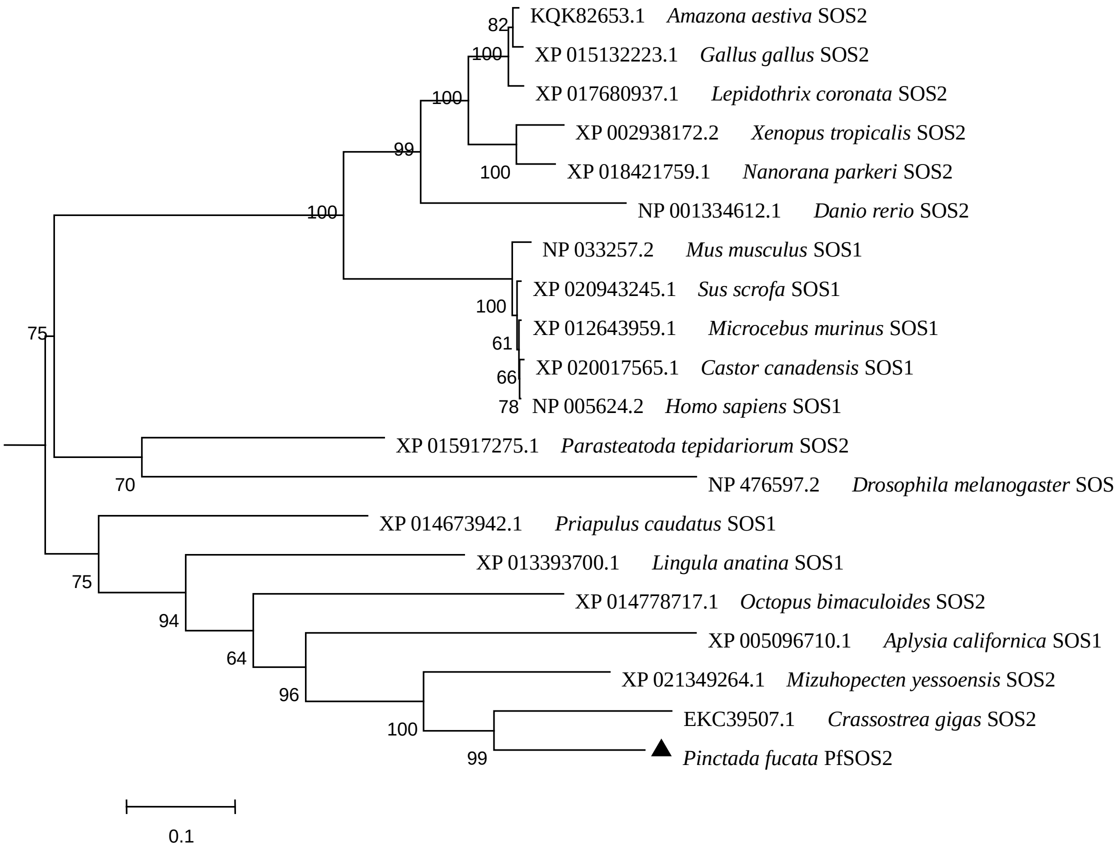
KQK82653.1 Amazona aestiva SOS2
XP 015132223.1 Gallus gallus SOS2
XP 017680937.1 Lepidothrix coronata SOS2
XP 002938172.2 Xenopus tropicalis SOS2
XP 018421759.1 Nanorana parkeri SOS2
NP 001334612.1 Danio rerio SOS2
NP 033257.2 Mus musculus SOS1
XP 020943245.1 Sus scrofa SOS1
XP 012643959.1 Microcebus murinus SOS1
XP 020017565.1 Castor canadensis SOS1
NP 005624.2 Homo sapiens SOS1
XP 015917275.1 Parasteatoda tepidariorum SOS2
NP 476597.2 Drosophila melanogaster SOS
XP 014673942.1 Priapulus caudatus SOS1
XP 013393700.1 Lingula anatina SOS1
XP 014778717.1 Octopus bimaculoides SOS2
XP 005096710.1 Aplysia californica SOS1
XP 021349264.1 Mizuhopecten yessoensis SOS2
EKC39507.1 Crassostrea gigas SOS2
Pinctada fucata PfSOS2
82
100
100
99
100
100
100
61
66
78
75
70
75
94
64
96
100
99
0.1

Supplementary Figure. 5

A

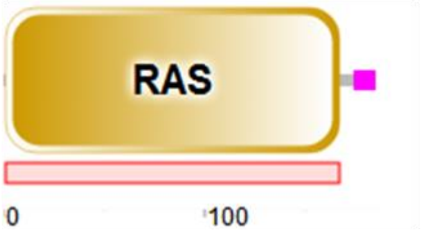

B

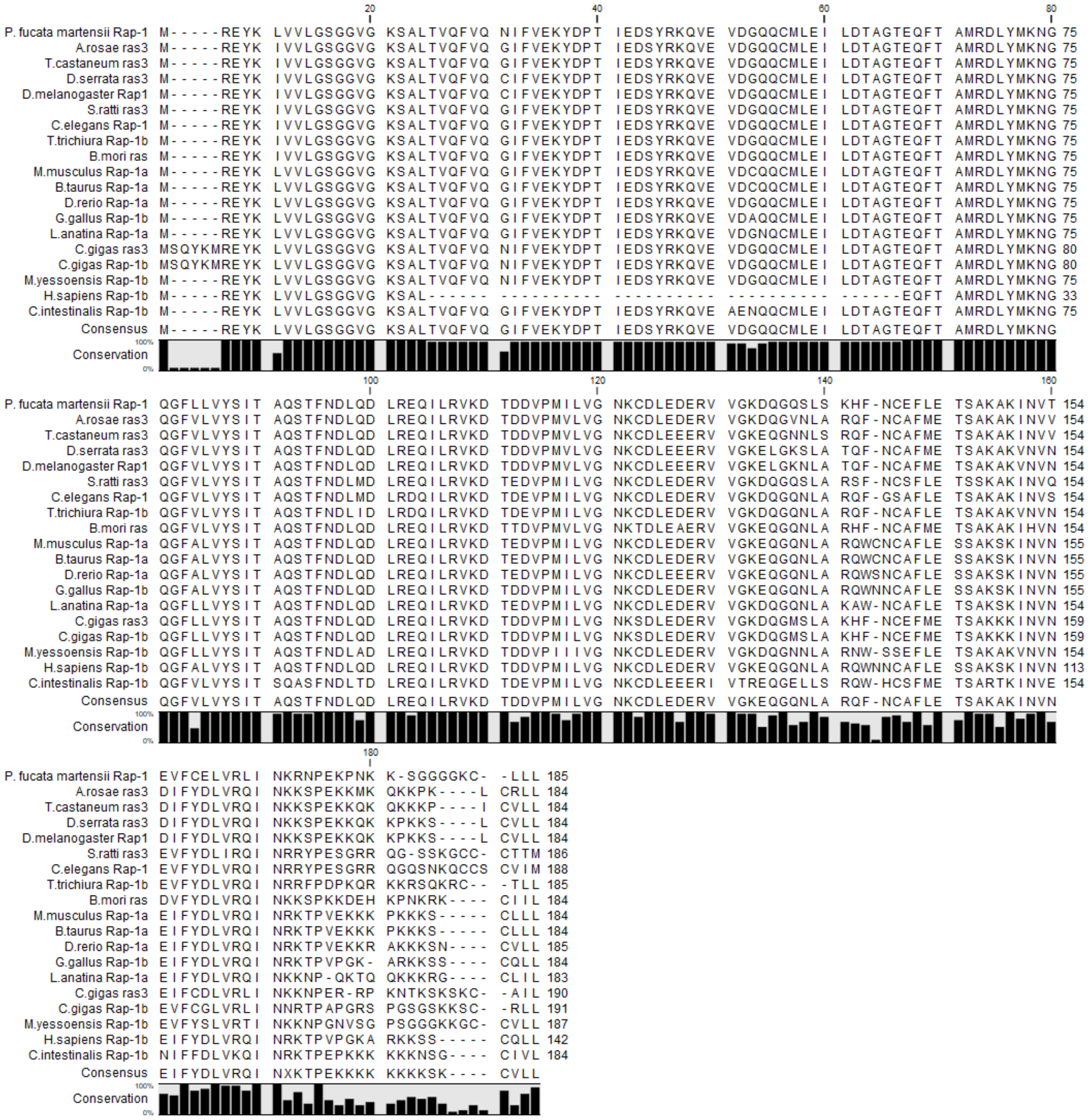

C

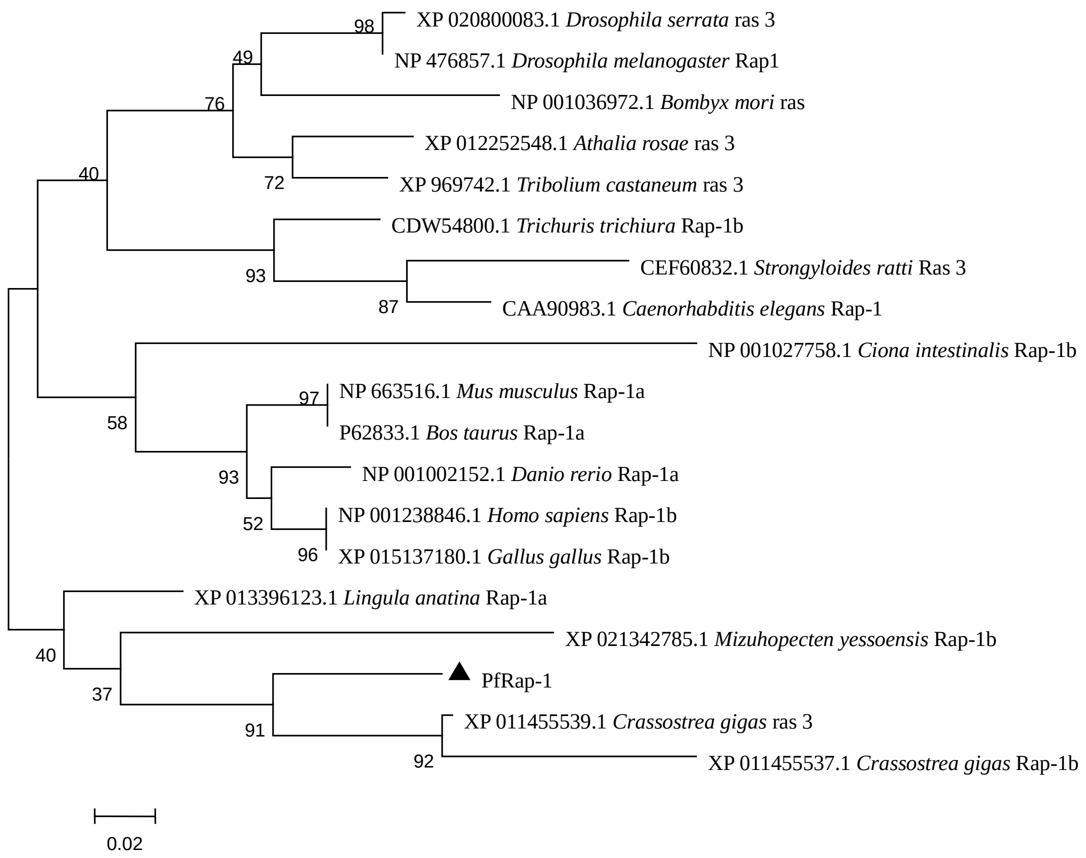
XP 020800083.1 Drosophila serrata ras 3
NP 476857.1 Drosophila melanogaster Rap1
NP 001036972.1 Bombyx mori ras
XP 012252548.1 Athalia rosae ras 3
XP 969742.1 Tribolium castaneum ras 3
CDW54800.1 Trichuris trichiura Rap-1b
CEF60832.1 Strongyloides ratti Ras 3
CAA90983.1 Caenorhabditis elegans Rap-1
NP 001027758.1 Ciona intestinalis Rap-1b
NP 663516.1 Mus musculus Rap-1a
P62833.1 Bos taurus Rap-1a
NP 001002152.1 Danio rerio Rap-1a
NP 001238846.1 Homo sapiens Rap-1b
XP 015137180.1 Gallus gallus Rap-1b
XP 013396123.1 Lingula anatina Rap-1a
XP 021342785.1 Mizuhopecten yessoensis Rap-1b
PfRap-1
XP 011455539.1 Crassostrea gigas ras 3
XP 011455537.1 Crassostrea gigas Rap-1b
98
49
76
40
72
93
87
58
97
93
52
96
40
37
91
92
0.02

Supplementary Figure. 6

A

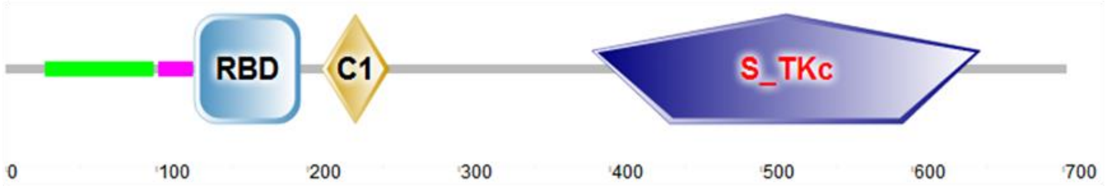

B

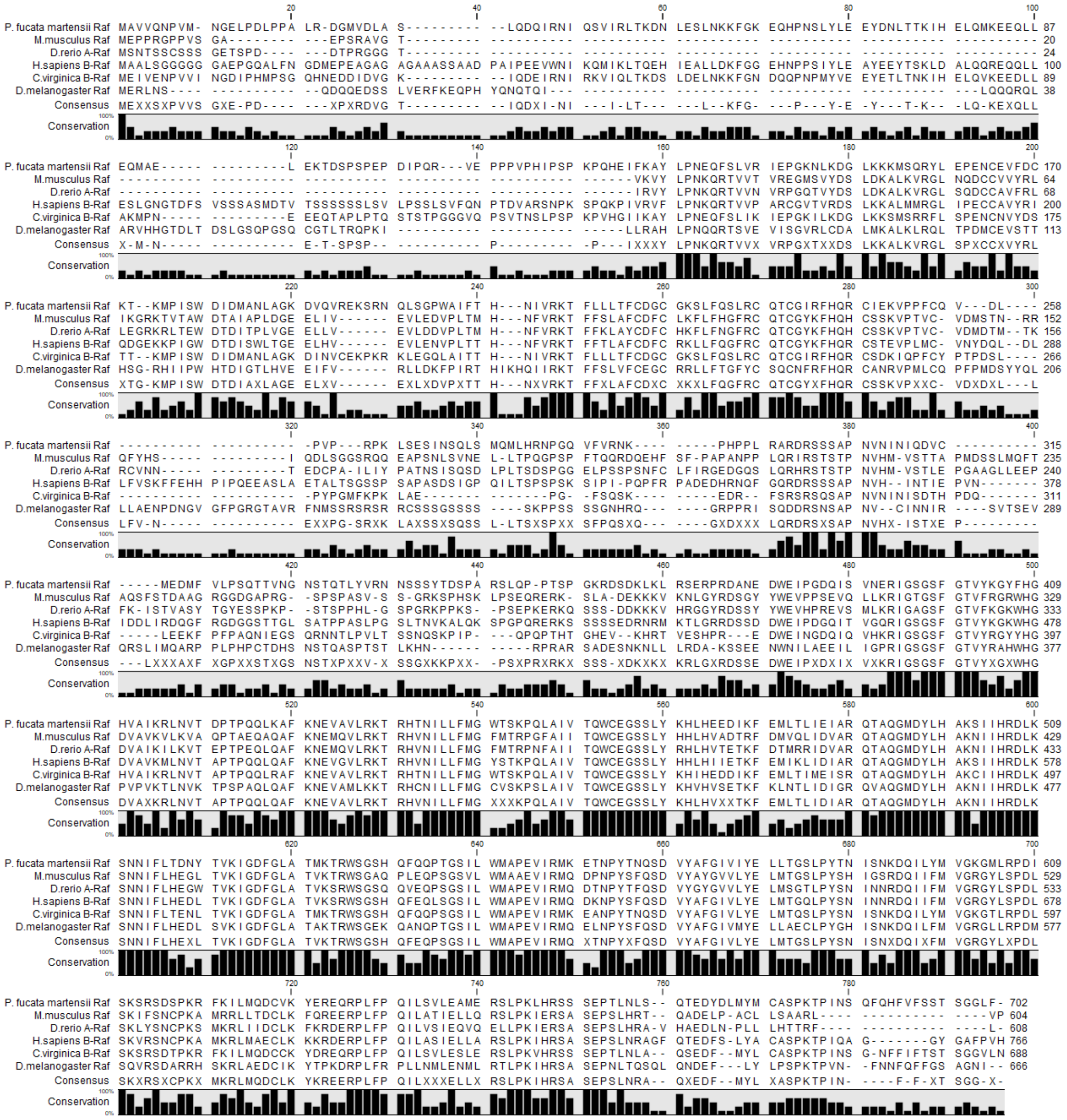

C

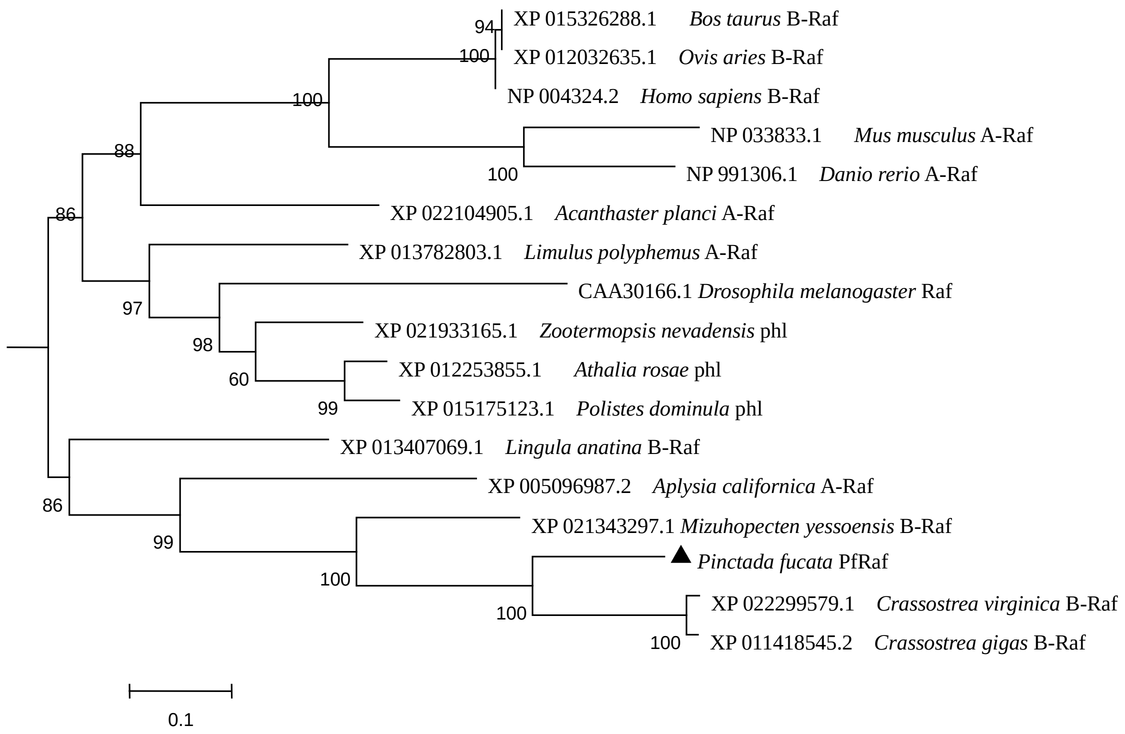
XP 015326288.1 *Bos taurus* B-Raf
XP 012032635.1 *Ovis aries* B-Raf
NP 004324.2 *Homo sapiens* B-Raf
NP 033833.1 *Mus musculus* A-Raf
NP 991306.1 *Danio rerio* A-Raf
XP 022104905.1 *Acanthaster planci* A-Raf
XP 013782803.1 *Limulus polyphemus* A-Raf
CAA30166.1 *Drosophila melanogaster* Raf
XP 021933165.1 *Zootermopsis nevadensis* phl
XP 012253855.1 *Athalia rosae* phl
XP 015175123.1 *Polistes dominula* phl
XP 013407069.1 *Lingula anatina* B-Raf
XP 005096987.2 *Aplysia californica* A-Raf
XP 021343297.1 *Mizuhopecten yessoensis* B-Raf
Pinctada fucata PfRaf
XP 022299579.1 *Crassostrea virginica* B-Raf
XP 011418545.2 *Crassostrea gigas* B-Raf
94
100
100
100
88
86
97
98
60
99
86
99
100
100
100
0.1

Supplementary Figure.7

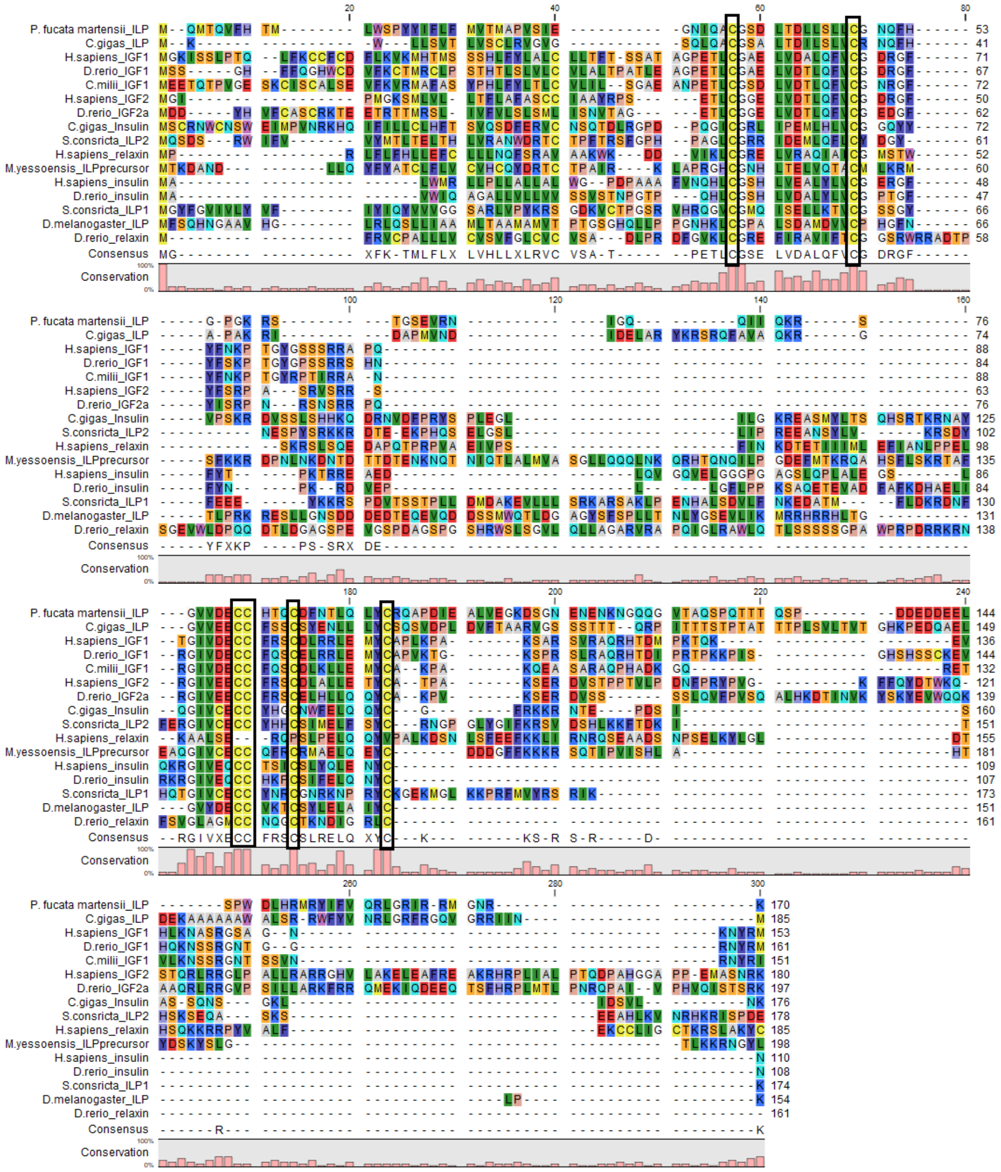

Supplement: Supplementary file 1 — Supplementary Information. [file 41598_2019_57329_MOESM1_ESM.pdf]
